# Insight into Structure Activity Relationship of DPP-4 Inhibitors for Development of Antidiabetic Agents

**DOI:** 10.3390/molecules28155860

**Published:** 2023-08-03

**Authors:** Vishal Mathur, Ozair Alam, Nadeem Siddiqui, Mukund Jha, Ajay Manaithiya, Sandhya Bawa, Naveen Sharma, Sultan Alshehri, Prawez Alam, Faiyaz Shakeel

**Affiliations:** 1 Medicinal Chemistry and Molecular Modelling Lab, Department of Pharmaceutical Chemistry, School of Pharmaceutical Education and Research, Jamia Hamdard, New Delhi 110062, India; vishalmathur3131@gmail.com (V.M.); nsiddiqui@jamiahamdard.ac.in (N.S.); jhamukund508@gmail.com (M.J.); ajaymanaithiya@gmail.com (A.M.); sbawa@jamiahamdard.ac.in (S.B.); 2 Division of Bioinformatics, Indian Council of Medical Research, New Delhi 110029, India; naveen.sharma83@gov.in; 3 Department of Pharmaceutical Sciences, College of Pharmacy, AlMaarefa University, Ad Diriyah 13713, Saudi Arabia; 4 Department of Pharmacognosy, College of Pharmacy, Prince Sattam Bin Abdulaziz University, Al-Kharj 11942, Saudi Arabia; prawez_pharma@yahoo.com; 5 Department of Pharmaceutics, College of Pharmacy, King Saud University, Riyadh 11451, Saudi Arabia; faiyazs@fastmail.fm

**Keywords:** DPP-4 inhibitors, SAR, heterocyclic scaffolds, bio-activity

## Abstract

This article sheds light on the various scaffolds that can be used in the designing and development of novel synthetic compounds to create DPP-4 inhibitors for the treatment of type 2 diabetes mellitus (T2DM). This review highlights a variety of scaffolds with high DPP-4 inhibition activity, such as pyrazolopyrimidine, tetrahydro pyridopyrimidine, uracil-based benzoic acid and esters, triazole-based, fluorophenyl-based, glycinamide, glycolamide, β-carbonyl 1,2,4-triazole, and quinazoline motifs. The article further explains that the potential of the compounds can be increased by substituting atoms such as fluorine, chlorine, and bromine. Docking of existing drugs like sitagliptin, saxagliptin, and vildagliptin was done using Maestro 12.5, and the interaction with specific residues was studied to gain a better understanding of the active sites of DPP-4. The structural activities of the various scaffolds against DPP-4 were further illustrated by their inhibitory concentration (IC_50_) values. Additionally, various synthesis schemes were developed to make several commercially available DPP4 inhibitors such as vildagliptin, sitagliptin and omarigliptin. In conclusion, the use of halogenated scaffolds for the development of DPP-4 inhibitors is likely to be an area of increasing interest in the future.

## 1. Introduction

**Figure 1:**
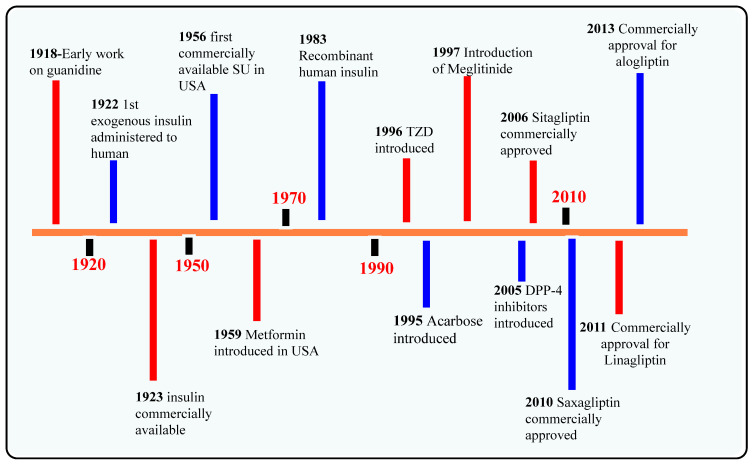
Work on DM during different timelines and demonstrating launch time of DPP-4 inhibitors.

Dipeptidyl peptidase-4 (DPP-4) is a serine protease present on the surface of diverse cell types, mostly in tissues of the kidney, liver, gastrointestinal, lymphocytes, placenta, and uterus, which not only helps in the modulation of biological activity of several peptide hormones like neuropeptides, cytokines, and chemokines but glucagon-like peptide-1 (GLP-1) significantly cleaves the protease chain specifically from proline and alanine amino acid residue present at N-terminal position which further leads to the inactivation of the GLP-1 (is one of the incretins which helps in regulation of blood homeostasis) [1,2]. DPP-4 helps in degrading incretins like GLP-1 and glucose-dependent insulinotropic polypepide (GIP), majorly responsible for glycaemic control by maintaining insulin secretion & reducing the secretion of glucagon from alpha-cells [3,4]. Apart from triumph in developing several potent drugs used in therapy for diabetes mellitus (DM) by shielding GLP-1 degradation, DPP-4 inhibitors could be much more beneficial in a broader spectrum of indications [4]. DPP-4 cleaves the intact GLP-1_7-36_ to GLP-1_9-36_ of alanine or proline (amino acid in GLP-1), and within 1–2.5 min, DPP-4 protease becomes rapidly inactive (which is extensive and fast). Developing DM based on different timelines and demonstrating the launch time of DPP-4 inhibitors are given in Figure 1. 

Data gathered from the international diabetes federation (IDF), about 425.0 million people had diabetes in 2017, and it hypothesized that numbers could varies as high as up to 700 million before 2045. DPP-4, also known as “CD26,” is a crucial element for virulence [5]. Diabetes is of chronic metabolic disorder that is blooming broadly every year, affecting millions of people around the globe. In 2006 first DPP-4 inhibitor was familiarized, signifying that they have the potential needed to treat diabetes and imitates lesser side effects, are highly potent, long-acting, and with a bioavailability of >90% & hence oral anti-hyperglycemic inhibitors got the focus of researchers and industry to work on [6]. Dipeptidyl peptidase-IV is one of the hit target proteins to treat type-2 diabetes and also for maintaining the glycemic level of blood. Modification in lifestyle & metformin is recommended as first-line therapy to treat type-2 diabetes mellitus [7]. Type-2 diabetes mellitus (T2DM) is mainly distinguished by insulin deficiency & insulin resistance; the main objective of treating T2DM is to sustain glycosylated hemoglobin levels below 7%. It has been identified that DM could lead to an increase in the fate of other diseases like cardiovascular, neuropathy, nephropathy, stroke, and hypertension [8]. Apart from the risk factors cited above, DPP-4 inhibitors indicate a benefit in cardiovascular safety by protecting the heart and preventing exposure to several adverse events and mortality in T2DM [9]. Several other incretins help in maintaining glycemic control in blood, like GLP-1, GIP-1, G-protein coupled receptors (GPCR) and fibroblast activation protein (FAP) [2,9,10].

**Figure 2:**
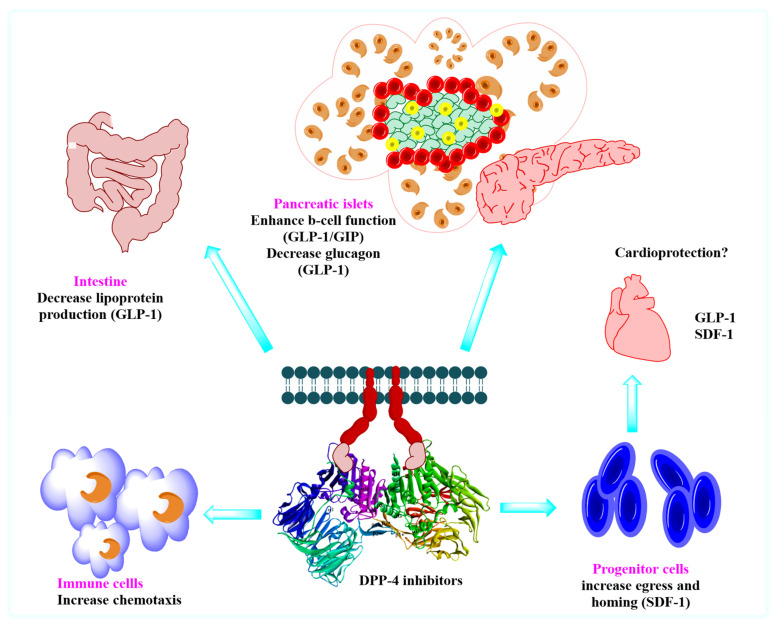
DPP-4 protease (PDBID:1X70) activity inhibition using DPP-4 inhibitors which also induces various biological actions in peripheral tissues.

Numerous “gliptins” have been synthesized in laboratories by researchers that inhibits DPP-4 and breaks off the cleaving step of amino acids from the N-terminal, and act as an agonist for incretins in maintaining glucose hemostasis; some of the marketed drugs available are alogliptin, saxagliptin, sitagliptin, vildagliptin, linagliptin and anagliptin (Figure 1). Drugs hold a different nucleus moiety which demonstrates contrast in physical and chemical properties, but also observed in seldom studies that 5th and 6th membered rings like piperazine, piperidine, pyrazine, xanthine, pyrrolidine, thiazolidine and thiosemicarbazide, bicyclic ring, and adamantane are some commonly used scaffolds that has shown anti-diabetic activity by amplifying incretin effects. Development of small molecules-based approaches emerging to treat Type-II diabetes, like incorporating 2(S)-cyanopyrrolidine scaffold into a molecule, has been used often & has become a key intermediate for the synthesis of DPP-4 inhibitors [11]. DPP-4 inhibitors might reduce renal outcomes. Several pieces of evidence denote that the second to third class of drugs are associated with rising risk factors like blood pressure (BP) [12,13]. Presently, several classifications of drugs have been already on the market for treating type-1 and type-2 diabetes having standard pharmacological actions including sulphonylurea (insulin secretagogues) [14], glinides (repaglinide, nateglinide) [15], biguanides (metformin) [16], glitazones (rosiglitazone, pioglitazone) [17], α-glucosidase inhibitors (miglitol, acarbose) [18,19] and thiazolidinediones (insulin sensitizers) [8,20]. Quinazolinone & its analogs have supported themselves as an effective compound in medicinal chemistry in developing & designing bioactive compounds [21]. The nitrile group is used more often as an effective pharmacophore in planning the number of DPP-4 inhibitor drugs [22,23]. DPP-4 inhibitors increase the bioavailability of incretins like GIP & GLP-1 [11] (Figure 2).

**Figure 3:**
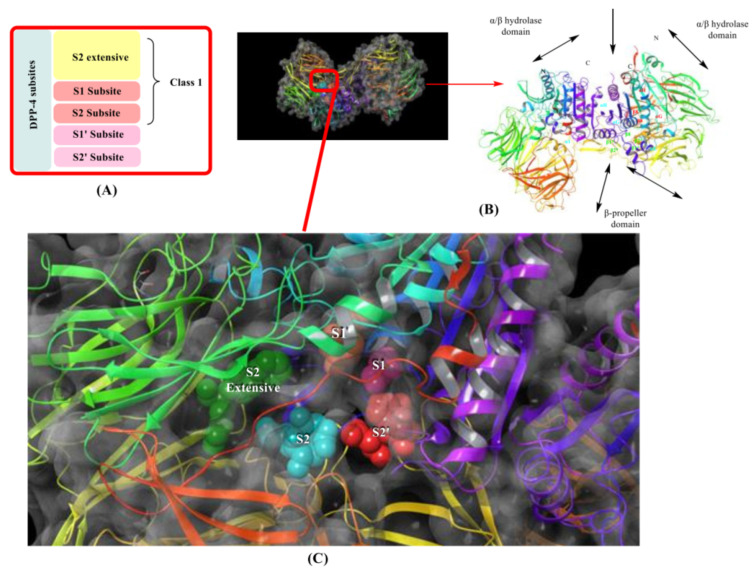
(**A**) DPP-4 enzyme sites (**B**) Structure of DPP-4 homodimer, the structure is prepared by using Pymol (**C**) illustrates subsites in the protein structure (PDBID:1X70) in CPK representation form.

Mentioned above are oral agents to be taken once or thrice per day and some exceptions to be administered once a week [bydureon-(i.v)]. They help in reducing fasting and postprandial hyperglycemia, weight remains neutral, and have a lower risk of hypoglycemia. Currently, they are being used with metformin as in combination therapy but have shown efficient pharmacological actions if taken as monotherapy [24]. Discussed classes of compounds have some frequent side effects like edema, weight gain, digestive related and other complications like cardiovascular diseases, neuropathy, nephropathy, hearing impairment, foot damage, Alzheimer’s diseases, obesity, and most vital is hypoglycemic episodes, whereas so-called “gliptins” does not share most of these side effects. In this context, we will intensify the structure activity relationship (SAR) DPP-4 inhibitors, synthesis, and IC_50_ of developed compounds for treating type-2 diabetes [25]. DPP-4 helps in the hydrolysis of incretins which prohibit the functions of hormones; several clinical trials and in vivo experimental animal models postulate that DPP-IV inhibitors have demonstrated high therapeutic potential in long-term therapy of T2DM decreases diseases progression and are being used in treating hyperglycemia [25,26]. DPP-4 consists of 766 amino acids and recognizes peptides having proline and alanine at the N-terminal position; crystal structure of DPP-4 forms a homodimer (chain-A and chain-B), it consists of three main domains (i) cytoplasmic, (ii) transmembrane and (iii) extracellular domain (Figure 3B).

The extracellular domain is further classified into two types: catalytic and 8-bladded β-propeller domain, DPP-IV has five subsites (binding sites) S_1_, S_2_, S_1_′, S_2_′, and S_2_ extensive. Interaction of ligand with S_1_ and S_2_ subunits is mandatory for inhibitory activity, whereas additional interaction with S_1_′, S_2_′, or S_2_ extensively increases the potency up to 4–5 times fold in DPP-4 inhibition. The S_1_ pocket of DPP-4 is founded to be very hydrophobic; teneligliptin has fivefold activity higher than in comparison with sitagliptin because it forms H-bond with S_1_, S_2,_ and S_2_ extensive subsites with DPP-4 (Figure 3A) [26,27,28]. Determining the diagram mentioned below states that the drugs are classified into three classes differentiating each class based on interacting with significant subsites (Table 1). Observing that the more the interaction of the drug with DPP-4 subsites better the resolution of IC_50_, with a higher potency of bioavailability. This leads us to design such molecules which could tend to form a higher number of interactions with DPP-4 protease subsites; different scaffold contributes their role in the interaction & potency of composed several halogen groups helping in the formation of Hydrogen-bond [28,29,30].

**Figure 4:**
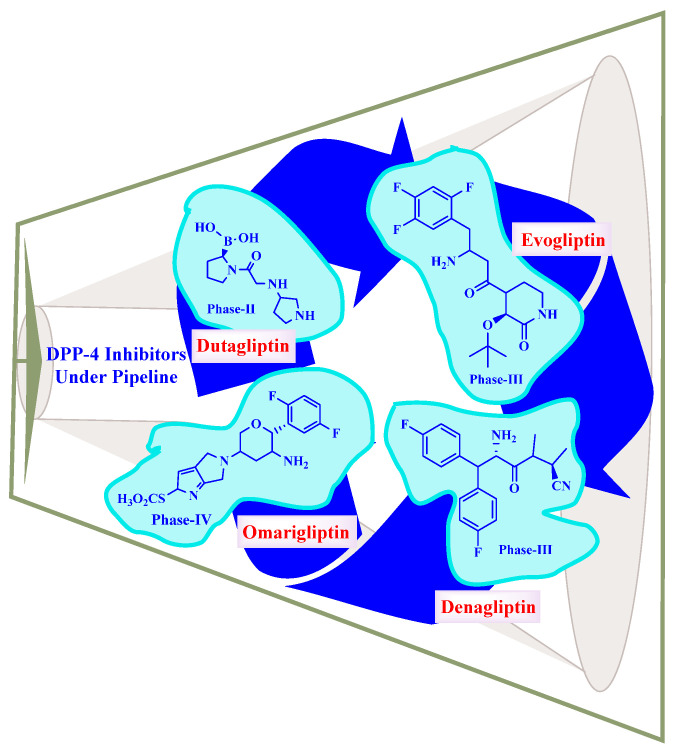
Novel DPP-4 inhibitors under clinical trial pipeline.

Inhibitors were categorised in several classes based on the binding of inhibitors to the subsites present, such as sitagliptin and teneligliptin were categorized in class 1 as it binds with with S1, S2, and S2 extensive subsite, those binding to S1′, S2′, S1 and S2 (alogliptin and linagliptin) were categorized in third class whereas, inhibitors like vildagliptin and saxagliptin were ranked in class second as they binds at S1 and S2 subsites only (illustrated in Figure 3). Interaction of named drugs such as vildagliptin, sitagliptin, saxagliptin are showed in Figure 4. The first class of drugs (e.g., vildagliptin and saxagliptin) founded to be interacting majorly with S_1_ and S_2_ subsites, cyannopyrrolidine moiety interacts with S_1_ whereas hydroxy adamantyl interacts with the S_2_ subsite. On the other hand, the second class of drugs binds by forming an additional subsite in comparison with the first class. Linagliptin binds with four subsites, including S_1_, S_2_, S_1_′, and S_2,_ yielding 8-fold higher activity than Alogliptin. Alogliptin finds to be binding with only three subsites, i.e., S_1_, S_2_, and S_1_′. Moreover, 3rd class, which holds teneligliptin, is a marketing DPP-4 inhibitor because of the pentacyclic ring. Teneligliptin has five times higher activity than sitagliptin because of the presence of a J-shaped anchor-lock domain and a stronger covalent bond with DPP-4, also an additional bond with S_2_ extensive subsite [22]. Development of dipeptidyl peptidase was done based on a gut-derived glucagon-like peptide that is one among the anti-diabetic hormone which increases the ability of secretion of insulin and inhibit glucagon secretion and helps in preventing the inactivation of GLP-1 hence a hike in circulation level of GLP-1 could be seen enhancing anti-diabetic action [24]. Tyr662, Tyr547, Glu205, Glu206, Phe357, Arg358, Ser630, Ser631, Asn710, Val207 are some amino acid residues with which dipeptidyl peptidase-4 inhibitors form covalent/H-bond or π-π interaction for blocking DPP-4 activity.

## 2. Mechanism of DPP-4 Inhibitors

**Figure 5:**
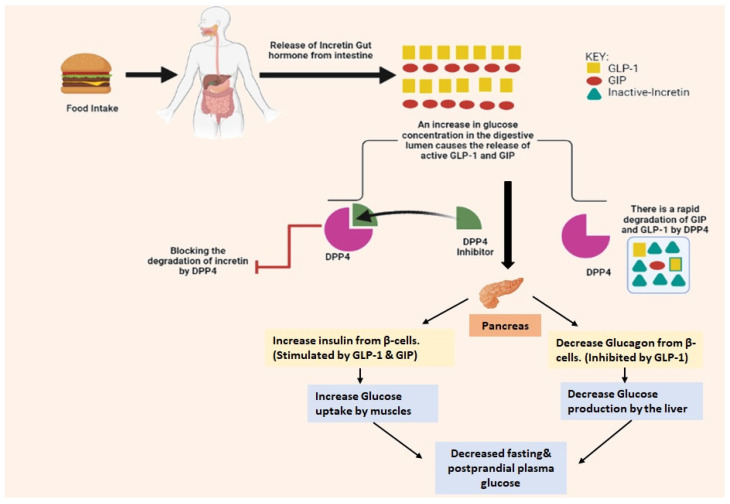
Pleiotropic mechanism of DPP-4 inhibition.

As depicted below in Figure 5, incretins like GLP-1, GLP-2, and GIP work upon their own GPCR present on the beta-cells membrane to trigger adenylyl cytase & further help in the generation of cyclic adenosine monophosphate (cAMP), which is also responsible for exocytosis of insulin. GLP-1 and GIP incretins get quickly inactivated by a capillary endothelial enzyme called di-peptidyl peptidase-4 [24,31]. Moreover, DPP-4 inhibitors such as vildagliptin, sitagliptin, and saxagliptin are mainly responsible for the increase in suppression of DPP-4 action and further accentuate insulin response against glucose hike via ingesting meal & also accentuate postprandial glycemia. It is possible that the activity of DPP-4 in peripheral blood may not be the only factor influencing glucose levels; it is also possible that the activity of DPP-4 in tissues, through the plasma within those tissues, could have a significant impact as well. The inhibition of DPP-4 may be responsible for lowering glucose levels almost as much as GLP-1 receptor agonists, which are more directly responsible for the activation of GLP-1 receptors [32,33,34].

## 3. Development of DPP-4 Inhibitors

Substrate-based DPP-4 inhibitors and non-substrate-based DPP-4 inhibitors refer to drugs used to treat diabetes. Substrate-based DPP-4 inhibitors are drugs that bind to the active site of the enzyme, inhibiting its activity and leading to increased levels of GLP-1. Non-substrate-based DPP-4 inhibitors are drugs that bind to the allosteric site of the enzyme, also inhibiting its activity and leading to increased levels of GLP-1. Both types of inhibitors can be used to treat type 2 diabetes as they can help the body to better control blood sugar levels [35].

### 3.1. Substrate-Based DPP-4 Inhibitors

Designing of DPP-4 inhibitor drugs is usually done using two basic methods (i) substrate based structure synthesis and (ii) non-substrate-based structure synthesis. Most of the compounds synthesized using substrate-based inhibitors approach, these compounds are proline mimetic, which occupy S_1_-pocket, P_2_-substituent of drug occupies S_2_-pocket whereas P_1_-substituent occupies S_1_-pocket of DPP-4 protease, and their basic structure interacts via either forming a covalent or non-covalent bond [36]. For instance, cyanopyrrolidines show two interactions: the first interaction is of the nitrile group, which forms reversible covalent bonds with catalytically active Ser630 (serine hydroxyl group and the other one is with a protonated amino group which forms a hydrogen bond with a negatively charged region of protein residues Glu205, Glu206 and Tyr662 surface (Figure 20) [35,37,38].

### 3.2. Non-Substrate Based DPP-4 Inhibitors

**Figure 6:**
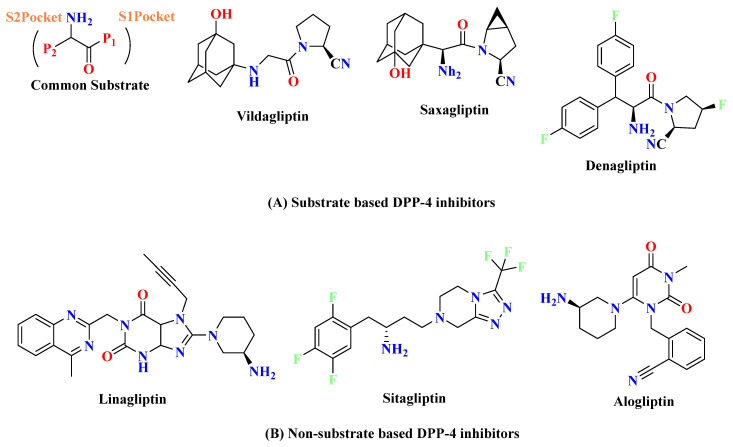
Substrate and non-substrate based DPP-4 market approved inhibitors.

Non-substrate compounds typically have an aromatic ring that occupies the S_1_-pocket of DPP-4 protease. Like proline mimetic-designed molecules, they don’t take after the dipeptides nature of DPP-4 substrates. Due to lack of selectivity, Merck stopped working on α-amino acid & started focusing on β-amino acids, which light up several rings like piperazine and triazolopiperazine. Pyrimidinedione has better metabolic stability & shown potent, selective, and bioavailable DPP-4 inhibitor known as alogliptin [39,40]. Since after development of sitagliptin which was approved by the U.S Food and drug administration (FDA) in 2006, nine DPP4 inhibitors are already available in the market and have shown great efficacy with lower toxicity when compared with existing therapies (Figure 6). Meanwhile, a newly developed potent class of antihyperglycemic drugs emerged out as selective DPP4 inhibitors with biaryl scaffolds moiety. These include derivatives of 4-phenylbenzimidazole, 4-phenyl-1,2-dihydroisoquinolin1-one, 4-phenylquinoline, 5-phenylpyridopyrimidinedione, 7-oxo-4-phenylpyrrolopyridine, 5-phenylimidazo- [1,2-a] pyrimidine, and phenylpyridine [35,40].

## 4. Highly Efficient Schemes to Synthesize Compounds

### 4.1. Sitagliptin

**Figure 7:**
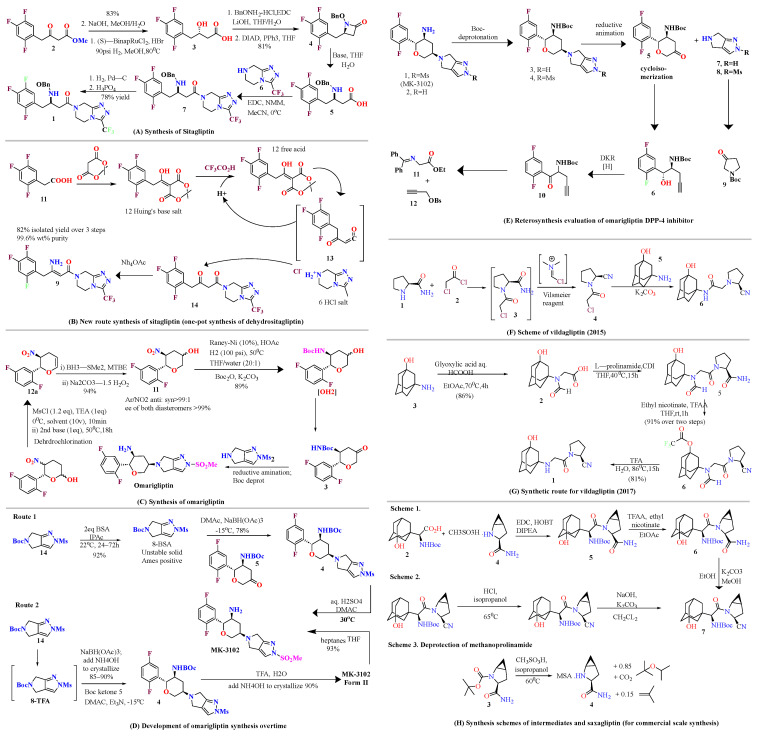

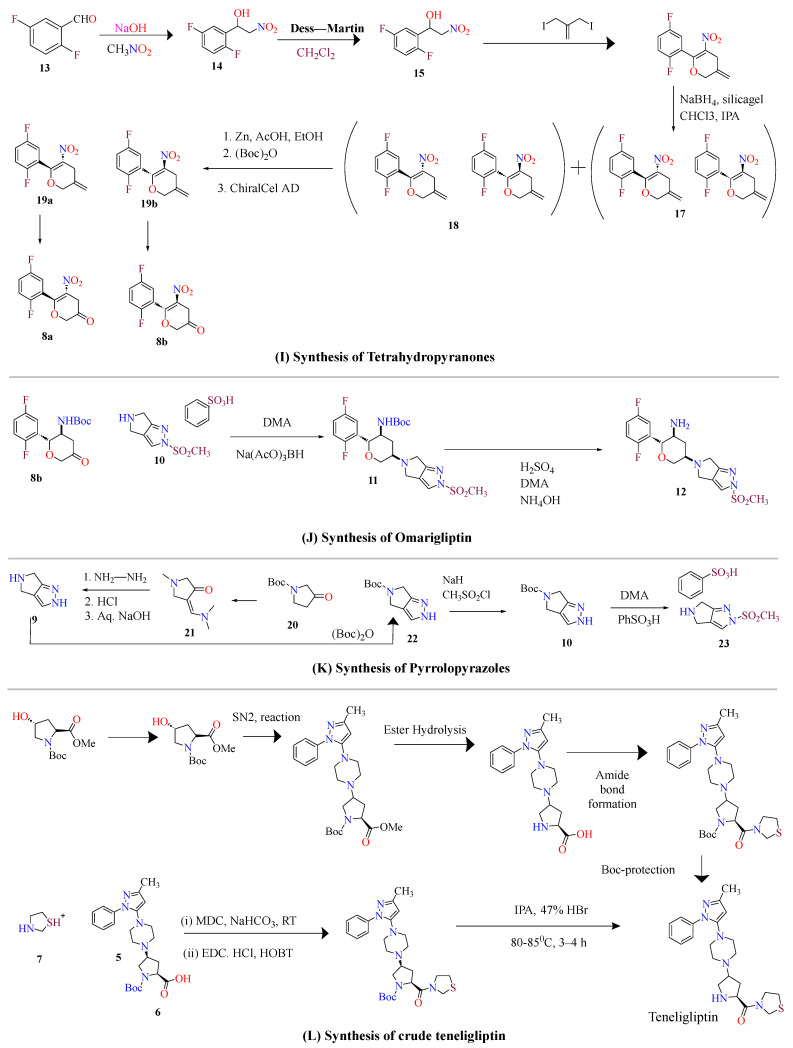
(**A**–**H**, **I**–**L**) General Scheme/Synthesis of market-approved DPP-4 inhibitor drug.

Synthesis of highly efficient, potent, and selective sitagliptin for treatment of T2DM were developed. Dehydrositagliptin intermediate is prepared by 2 step process in one pot and isolated in 82% yield with 99.6% wt. purity. Hydrogenation of highly enantioselective dehydrositagliptin, with 0.15 mol% of Rh(I)/^t^Bu JOSIPHOS, yielded sitagliptin which is further isolated in its phosphate salt with better chemical and optical purity. Mentioned synthesis has reduced total waste from 250 kg to 50 kg, which is nearly five-fold, and completely eliminated aq. Waste streams [41]. As illustrated in Figure 7A, synthesizing sitagliptin by using an earlier scheme produced 5X waste when compared with the new route (Figure 7B); total waste was analyzed as well as aq. Waste stream. Compound **1** isolated in its phosphate monohydrate form from aq. propyl alcohol (PrOH) with 96% yield with a purity level of >99.9%, which is nearly perfect. The first generated scheme meant to prepare large quantities (>100 kg) of sitagliptin in favor to support clinical studies and safety, despite that the overall yield of the compound was as high as 52% yield including multiple-step reaction and isolations led to producing a larger quantity of waste. Specifically, 1-ethyl-3-(3-dimethylaminopropyl) carbodiimide (EDC) coupling-Mitsunobu sequence engaging to convert hydroxyl group of 3 to masked amino group of 4 compounds was the major reason for waste output and poor atom economy too [41]. 

### 4.2. Omarigliptin

Synthesis of highly efficient asymmetric tetrahydropyranol intermediate are explained [42,43,44]. Development of metal-free and the protecting group was achieved via approaching asymmetric Henry reaction and one pot intro Michael-localization-dehydration via the process. Synthesis of a given complex intermediate was done in four simple linear steps and by utilizing simple starting materials [42]. Using 2,5-difluorobenzaldehyde as substrate, numerous marketed available ligands were surveyed using high-throughput experimentation techniques and tools. Cu(OAc)_2_ in combination form with mentioned ligands formation of desired arylnitroethanol **C** (in 83%ee and 85–90% yield) was catalyzed under mild conditions using DABCO (as the base) and solvent (ethanol). For the proposed one-pot process to dihydropyran **A** by coupling **C** with acrolein, initial nitro-Michael-Localization sequence reactions are based on literature precedent using acid (pivalic acid, boric acid, or formic acid) and secondary amine (diphenylprolinol trimethylsilyl ether or Et2NH). Utilizing these conditions, the reaction rate was leisurely slow, requiring about 3–4 days for completion and 2 eq. of acrolein to catch 95% in converting lactol **B** into variable yield (60–80%). Treating lactol with 1.2 eq. of MsCl and 2.4 eq of triethyl amine (TEA) for 10 min and 2 h at 50 °C gives **A** a yield of 52% [42]. Using 2nd base (2,4,6-collidine) and methyl cyanide (MeCN) as a solvent increases the assay yield of **A** (which is starting material for the synthesis of omarigliptin) by 97% (Figure 7C,D) [42]. Convergent synthesis of DPP-4 inhibitor omarigliptin (MK-3102) for commercial manufacture is represented and developments implemented in it. The desired product is assembled by using diastereoselective reductive amination of pyranone with pyrazole via deprotecting a Boc group. It has been observed that the whole synthesis relies on 3 Ru-catalyzed reactions, (i) bis homopropargylic alcohol cycloisomerization to dihydropyran, (ii) DKR reduction of racemic-α aminoketone to fit two contiguous stereogenic centers, (iii) pyranol oxidation to get desired pyranol via Ru-catalyzed oxidation [43]. Primary multi-kilogram (kg) synthesis delivery of omarigliptin is founded to be a captious evaluation of discovery chemistry, and it addressed regiochemical control over N-sulphonation of pyrazole moiety embedded with **C**. With 1:1 ratio of two N-sulfonated isomers was obtained using sodium hydride (NaH) & methanesulfonyl chloride (MsCl) for sulphonation of 13, necessitating chromatographic purifications [43]. Figure 7D was discovered by Merck Research Laboratories for omarigliptin (a highly functionalized long-acting inhibitor). Similar chemistry was adopted for synthesis in kilogram by methylation of **D** via deprotection of Boc. At the same time, this process was founded to be capable and productive but did not meet the potential for commercial manufacture. Vital improvisation was done to first generation Synthesis of Boc-ketone **E** to reduce the mass intensity, cost reduction, and enhancement in robustness. In context, it holds the development and evolution of an efficient scheme for the multi-kilogram scale of omarigliptin (Figure 7E) [43]. The synthesis of omarigliptin (MK-3102) is illustrated in diagrammatic schemes in Figure 7I. Synthesis of **12** is done by reductive amination of tetrahydrapyranone (B) in the presence of triacetoxyborohydride. Omarigliptin founded to be a potent DPP-4 inhibitor and found to be a competitive inhibitor with an IC_50_ of 1.6 nanomolar (nM) [45]. Synthesis of intermediates of tetrahydropyranone **A** and **B** are illustrated as follows. Nitroketone was synthesized by reacting an aldehyde with nitromethane with a catalytic amount using sodium hydroxide, resulting in oxidizing nitro-alcohol. Intermediate 10 (methylsulfonylpyrrolopyrazole) was synthesized from Boc-protected pyrrolopyrazole in presence of methane-sulfonyl chloride, sodium hydride & deprotection (Figure 7J–K) [45].

### 4.3. Vildagliptin

Synthesis of vildagliptin was done by utilization of Vilsmeier reagent (VR) [23]. Using this 3-step scheme for synthesizing vildagliptin, a powerful DPP-4 inhibitor was developed leading competition to other existing procedures. This approach demonstrates the method used for synthesizing vildagliptin by mitigating hygiene hazards and potential safety [23]. Castaldi et al. synthesized vildagliptin using efficient and concise scheme giving 63% yield. Using this novel 4-step scheme with an overall yield of 63% on a >100 g scale, it is developed from 3-amino-1-adamantanol, L-prolinamide, and glyoxylic acid in a four-step reaction with isolation of only 2 intermediates [46]. Mentioned 2017 scheme [46] is much more efficient than others, yielding 63% of vildagliptin with much lesser waste produced and reducing time for the occurrence of reaction as compared with Pellegatti and Sedelmeier reaction (Figure 7F,G) [23].

### 4.4. Saxagliptin

For commercial scale synthesis of saxagliptin, two unnatural amino acids (**2** and **3**) were used, as mentioned in the scheme below. Lead compound was synthesized after deprotection of **3** and was formed via amide coupling of methanoprolinamide (**4**) and **2 [44]**. The salt form of saxagliptin was shown to be stable in solution form, whereas synthesis of free base monohydrate was a challenging task because of the conversion of free amine to 6-membered cyclic amidine. Several modifications were made for the synthesis of saxagliptin, such as the synthesis of intermediate N-Boc-3-hydroxyadamantylglycine [44] (Figure 7H).

### 4.5. Teneligliptin

Illustrated below-designed scheme was developed for the commercial production of teneligliptin hydrobromide hydrate (**1**). To synthesize the compound new intermediates with higher reactive nosyl derivative through SN_2_ nucleophilic substitution, further isolation was done by desertification of the intermediate. Preparation of new intermediates was done under reaction conditions, and coupling reaction was used for amidation & also for deprotection of N-Boc were optimized for evaluation & controlling impurities for maintaining the quality of the compound. The procedure helped overcome long-time consumption, expensive reagents, and laborious approaches, including the isolation of intermediates (Figure 7L) [44].

## 5. SAR Activity of DPP-4 Inhibitors 

### 5.1. Tri-Fluorophenyl-Based Scaffold Compounds

**Figure 8:**
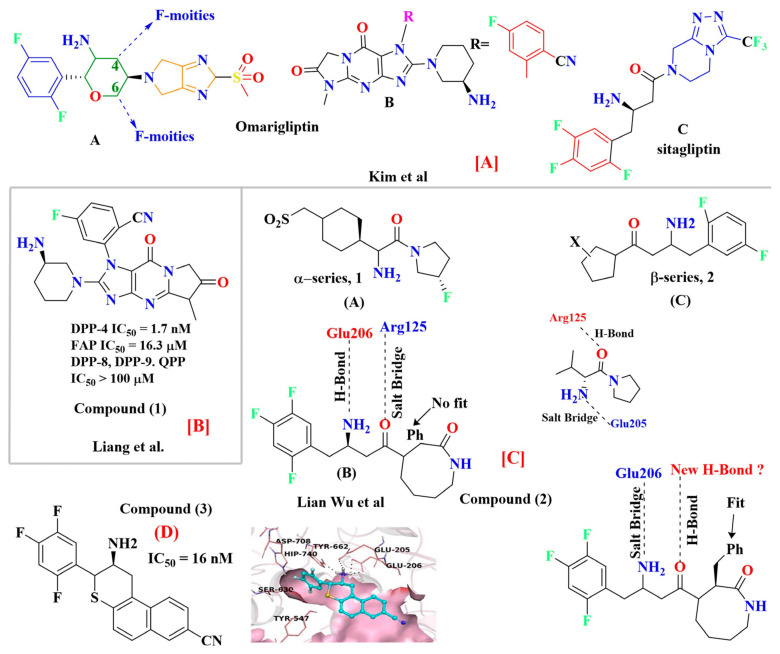
Structure of DPP-4 inhibitors: (**A**) omarigliptin and sitagliptin. (**B**) Compound (**2**) topmost potency against DPP-4 activity. (**C**) Compound (**A**–**C**) containing DPP-4 analogs. (**D**) Compound (**3**) containing DPP-4 design analogs [48,49,50].

Omarigliptin consists of a tricyclic ring (composed of di-fluorophenyl, tetrahydropyran, and pyrrolopyrazole ring attached to sulphonyl group) and is elongating as a once-weekly regimen. Fluorine exhibits magnificent properties like high electronegativity, lipophilicity, small atomic radius, and electrostatic interaction. Adding fluorine could improve pharmacokinetic and pharmacologic efficacy, membrane permeability, or metabolic stability. Substituting or adding fluorine at the 4th or 6th position of the tetrahydropyran ring can enhance the pharmacokinetic properties. To ensure the DPP-4 crystal structure is bounded with omarigliptin (prepared using QuickPrep in MOE), molecular operating environment (MOE) was used & R-vectors function was applied in MOE. To see several designed possible molecules, numerous groups, including the 4th and 6th position on tetrahydropyran moiety of omarigliptin were added (fluoro and trifluoromethyl groups were chosen as ideal units). Adding fluorine at the 4th and 6th position of the tetrahydropyran scaffold yields an IC_50_ of 3.07 nM, whereas CF_3_ at the 6th position gives 4.18 nM proving that the potency of the developed compound increases the potency by 4-folds (for orally and I.V both) and clearance rate was depressed too due to the presence of fluorine. Minimization of the salt bridge formation was done by substituting trifluoromethyl at position-6th instead of fluoro moiety, too far from the NH_2_ group [47]. Kim et al. state that 2,5-fluorophenyl derivates and 2,4,5-tri fluorophenyl exhibit an increase in potency of synthesized compounds by 5–7-fold as DPP-4 inhibitors. 2,4,5-tri fluorophenyl scaffold occupies mostly S_1_ hydrophobic pocket, and enhancement in potency is founded to be more than 3,4-difluoro analogs [48] (Figure 8A). Wu et al. synthesized a series of novel tricyclic compound derivatives, throughout which 17c (Figure 8B drawn below) is founded to be the most active potent drug with an IC_50_ of 1.7 nM against DPP-4 and FAP too. The structure was prepared from a mixture of regioisomers; adding only the cyano group on the benzyl ring improved potency by several folds yielding an IC_50_ of 10.6 nM, whereas adding 5-fluoro in 2-cyanobenzyl ring increases more potency exhibiting an IC_50_ of 1.7 nM. By retrieving this data it can be concluded that the cyano or fluoro group is vitally useful for the potency of the compound [49] (Figure 8B). Liang et al. speculated that fluorobenzyl and fluoropyrrolidine amide scaffold occupies the same hydrophobic pocket of the DPP-4 active site. Replacing fluorobenzyl moiety with fluoropyrrolidine decreases potency against DPP-4; as per results obtained, it is postulated that fortified similarity between both compounds is seen (fluoropyrrolidine amide moiety of α-series and fluorobenzyl β-series) & both holds a same active pocket in the DPP-4 enzyme active site. The Fluorobenzyl group of the β-series occupies a hydrophobic pocket and forms a salt bridge through an amide group of compounds with Glu205 and Glu206, whereas an H-bond is also speculated to forming between 2-fluorobenzyl group interacting with Asn710. It is envisaged that the CO group could also be used as substituting fluorine will retain the same result with similar potency [50] (Figure 8C). A new set of powerful DPP-4 inhibitors with dihydrobenzo[f] thiochromen scaffolds were designed and tested. Compound (**3**) binds to DPP-4 by way of the 2,4,5-trifluoro benzene group becoming embedded in the hydrophobic pocket of DPP-4 shaped by residues Val656, Tyr631, Tyr662, Trp659, Tyr666, and Val711. The nitrogen atom of the tetrahydro-2H-thiopyran-3-amine is well-positioned to form salt-bridge and hydrogen bond interactions with side chains of Glu205, Glu206, and Tyr662. Additionally, the naphthalene fragment gets involved in π-π stacking interactions with Phe357, while the electron-withdrawing CN can create a charge-reinforced hydrogen bond with the flexible Arg358. The introduction of small groups like OMe and Br, however, gave different results, implying that hydrophilic groups (-OMe) are preferable to hydrophobic ones (-Br) in the solvent region (Figure 8D) [51].

### 5.2. Adamantane Scaffold

**Figure 9:**
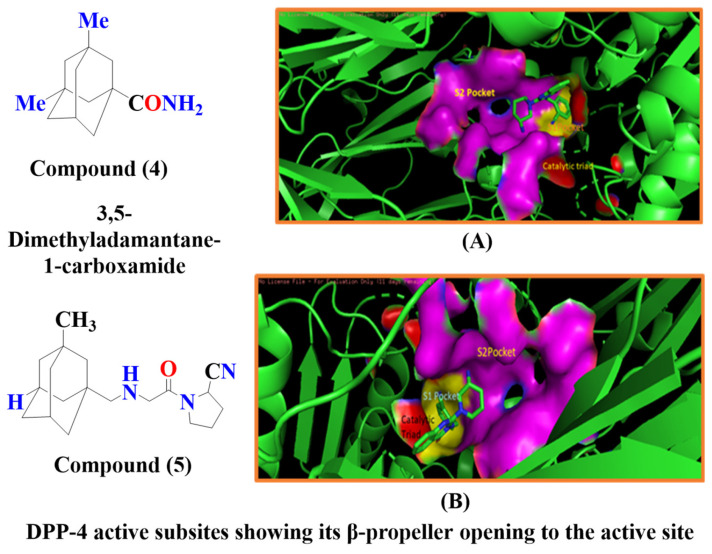
Structure of DPP-4 inhibitors: (**A**) Compound (**4**) containing DPP-4 active subsites (PDBID:1X70) showing its β-propeller opening to the active site. (**B**) Compound (**5**).

According to Al-wahaibi et al., inserting adamantane moiety in several molecules leads to yielding relatively higher lipophilic compounds as well as leads in the modulation of their therapeutic indices, and bioavailability and also exhibits a once-in-a-day pharmacokinetic profile. Adamantane scaffold (compound **4**) could affect a larger diversity of ailments; for instance, it is used in developing anticancer, antiviral, anti-TB, anti-hypertensive, and anti-hyperglycemic drugs (e.g., vildagliptin and saxagliptin) [52] (Figure 9). As per Spasov et al., Figure 8 states adamantane fragment is positioned similarly to vildagliptin and forms extensive hydrophobic interaction with residues of aromatic acids (Phe357 and Tyr666). Moreover, highlighted molecule (A) binds with Tyr547 residue and is implemented via p-electron alkyl bond interaction, whereas saxagliptin interacts via hydrogen bond (H-bond) between hydroxy (OH) groups. Identical interaction of vildagliptin against DPP-4 is also seen; the amide group of 3,5-dimethyl adamantane-1-carboxamide forms H-bond with Glu205 & with Glu206 and is also oriented in other directions too [53]. DPP-4 protease is constructed by five subsites, S_2_ subsite buildup of several amino acids like Arg125, Arg358, Arg669, Phe357, Glu205, and Glu206, shown in the figure in magenta color, whereas the catalytic triad is composed of Ser630, Asp708, Asn710, His740 amino acid residues highlighted by red color. S_1_ subsite is consisted of Val656, Trp659, Tyr662, Tyr666, Tyr631, Val711, glittered by yellow color. S_1′_ subsite constructed by Tyr666, Tyr547, Tyr631, Ser630, Phe357, Pro550 whereas, S_2′_ is made up by Ser630, Trp629, His630 and Tyr547 in cyan [54] (Figure 9A,B). Pannier et al. presented two new rigid synthetic scaffolds of adamantane; these scaffolds were designed to assemble multivalent binders for cell surface epitopes. The adamantane nucleus holds three carboxlic acid (COOH) groups in well-defined tripodal geometry to conjugate targeted ligands. Adding an amino group at the 4th bridgehead position provides a flexible linker for binding effector molecules such as cytotoxin without hampering the cell binding process. Multivalency is one of the most known phenomena to enhance the specificity and affinity of the ligand. The binding affinity of the developed multivalent molecules could be affected by orientation, size, and number of active binding sites as well as by shape and flexibility of the moiety (Figure 9A). The Figure 9 results illustrate that compound (**5**) is efficient in lowering glucose levels and stimulating insulin secretion. This can be attributed to the presence of the P-2 site valine, which fits into the catalytic binding site without any particular interactions with DPP-IV. Moreover, the role of the 2(S)-cyano group and the P-1 site pyrrolidine ring in our N-substituted glycine series implies that inhibitors similarly bind DPP-IV to valine pyrrolidine and DPP-IV (Figure 9B) [55].

**Figure 10:**
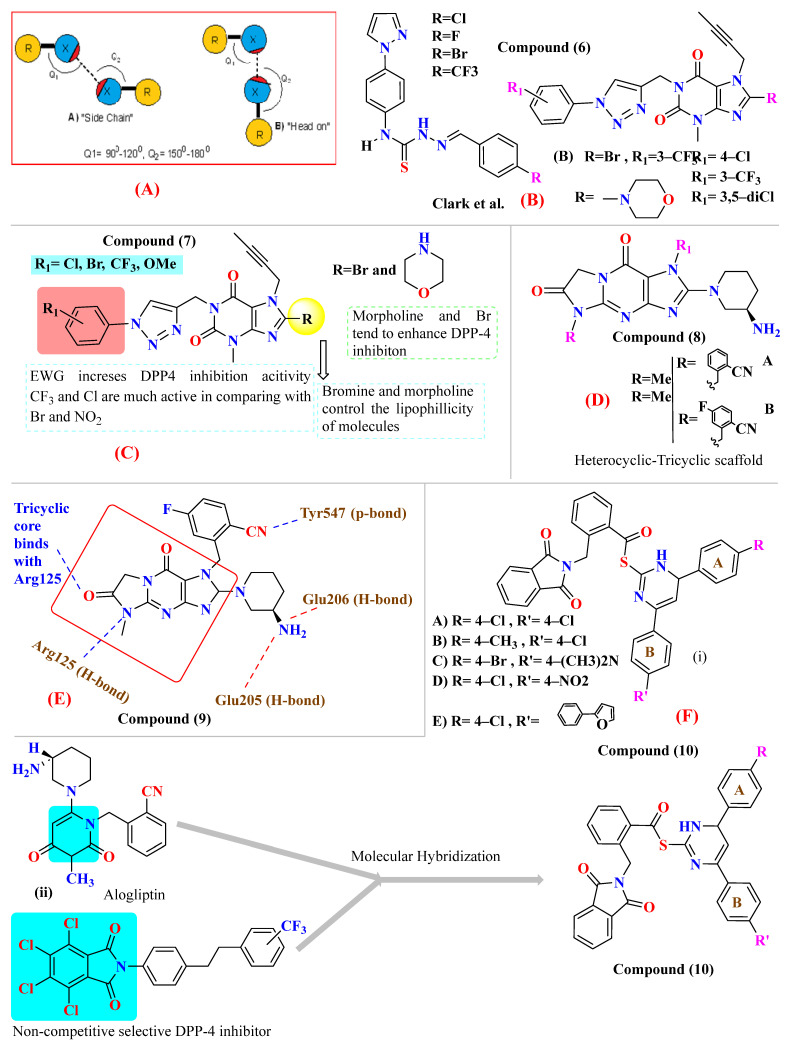
Structure of DPP-4 inhibitors: (**A**) Halogen binding with Electrophiles and nucleophiles in various fashions. (**B**) In vitro potency of inhibiting DPP-4 on substituting R with halogen atoms (Compound **6**). (**C**) SAR explains substituting R with halogen atoms (Compound **7**). (**D**) Novel tricyclic moiety compound **8**. (**E**) Compound (**9**) forming interaction with amino acid residues. (**F**) Design of Novel Hybrid synthesized DPP-4 inhibitor and their potent derivatives (Compound **10**) [56].

Protein-ligand interaction of halogen bonds was majorly the result of serendipitous discovery instead of rational design by giving examples where halogen bonding was exploited for lead optimization & identification. Halogens, specifically chlorine and fluorine are widely used in medicinal chemistry as they perceive hydrophobic scaffolds. Compounds holding halogen groups like chlorine, bromine, iodine, and fluorine can form direct interaction of type R-X
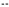
Y-R′, where X (halogen could act as Lewis acid) & Y can act as electron donor [56]. Wilcken et al. discover, using the natural bond order of CH_3_Cl (methyl halides), that nucleophiles contact Cl, Br, and F halogens with a “head on” fashion. In contrast, electrophiles approach in a “side chain” fashion. Because of the assembly of several possibilities of ligand-protein interaction, halogen bonding is a significantly suitable tool to improve compound specificities and affinities. Evaluation of halogen bonding strength could be done theoretically using quantum chemical mode calculations (e.g., QM-based evaluation) or experimental studies. Moreover, it is seen that halogen groups form halogen bonds with the carboxyl group (C=O) of amino acid residues, and its electrophilic nature enhances the efficacy of agonists. Adding just a halogen group can affect the effectiveness and potency of the compound, but substituting it on a particular ring could have much more efficacy to yield a much more potent compound [56] (Figure 10A). Introducing halogens in compounds has enhanced the potency of several classes of HIV reverse transcriptase inhibitors, hepatitis C virus NS3-Ns4A inhibitors, and α-4, β-2 nicotinic acetylcholine receptor, DPP-4 inhibitors and have also been exploited in the designing of anticancer drugs (Figure 10B) [40,54,55].

Derivates-based pyrazole incorporated thiosemicarbazone was designed and synthesized, demonstrating its potency based on substituting R as halogen. Substituting bromine at position R gives the most potent compound as a DPP-4 inhibitor with an effective IC_50_ of 1.266 ± 0.264 nM, comparing bromo-substitution is founded to be much more effective than sitagliptin which has an IC_50_ value of 4.380 ± 0.319 nM. Proving that introducing the bromo group at the para position of the benzylidene scaffold could enhance DPP-4 inhibiting potency, whereas substituting bromo with fluorine leads to a vital decrease in DPP-4 inhibitory activity. Moreover, adding CF_3_ (trifluoromethyl) at position R yields IC_50_ of 4.775 ± 0.296 nM, which in comparison to sitagliptin is much similar, indicating that the hike in DPP-4 inhibition properties could be because of the presence of CF_3_ at benzylidene moiety [57]. Improvement in selectivity and affinity of the mentioned compounds is because of trifluoromethyl and bromo group substitution at the para position, which has played a major role in DPP-4 inhibition. π- π interaction association of compounds with Arg358 is the reason for the stronger potency of the compounds; sitagliptin is also founded to form similar interaction with the same amino acid residue i.e., Arg358 and Tyr666; it is also disclosed that forming H-bond or salt bridge with Glu205 and Glu206 could also hamper the DPP-4 inhibition [57]. Substituting halogen groups at position R on benzene (R) and (R_1_) exhibited an enhanced inhibitory effect against DPP-4, and numerous substituents were added to the benzene ring. Adding morpholine at position-8 of xanthine moiety and CF_3_ group of benzene ring shows potent activity with an IC_50_ of 16.34nM whereas, substituting CF_3_ with dichloro also exhibited good potent activity against DPP-4 with IC_50_ of 29.87 nM moreover adding monochloride atom at para position founded to be reducing DPP-4 inhibition when compared with dichloro [(at meta-position (IC_50_ of 67.98 nM)]. Adding to the statement, adding electron donating groups (n-butyl, methoxy, etc.) at the benzene ring was found to decrease the potency of DPP-4 inhibitors, whereas substituting electron donating group (EDG) with electron withdrawing groups (EWG) like: bromo, and nitro) tended to enhance the activity, comparing the potency of the compounds within halogen substituting (Bromo to chloro or CF_3_) displays to see a reduction in inhibitory potency [58] (Figure 10 C,D). While developing tricyclic novel compounds, adding methyl at R and 2-cyano benzyl at R_1_ position enhances the inhibition potency against DPP-4 significantly with IC_50_ of 10.6 nM [comparing with alogliptin (IC_50_ 7.6 nM)]. Moreover, adding a fluoro atom, specifically at the 5th position of 3-cyano benzyl, enhances the compound’s activity by 10-folds with an IC_50_ of 1.7 nM. Furthermore, replacing the cyano group (with electron-withdrawing property) leads to a decrease in the potency of the further developed compounds. Replacing R with the ethyl group leads to having similar or decreased potency [49] (Figure 10E). These novel-developed tricyclic scaffold compounds were observed to interact similarly to alogliptin; the amino group forms interaction with Glu205 and Glu206 (glutamic acids), whereas the cyano group was analyzed to bind with Arg125 via H-bond. The tricyclic core of the compound was found to hoard against Tyr547 via π-bond (more extensive in comparison with alogliptin) (Figure 10F) [49,59]. Novel hybrid synthesized compounds D&E are founded to be most potent relative to other derivatives exhibiting IC_50_ of 0.51 and 0.66 nM. The potent DPP-4 inhibitor behavior of synthesized E is attributed to the electronic behavior of the furanyl group present in ring A along with the 4-Me2N group in ring B which facilitates H-bond formation with the active site of DPP-4. The mesomeric effect induced from 4-bromo substitution in ring A of C (hybrid compound) enhances DPP-4 inhibition activity in nM potency (IC_50_ 1.42 nM 4 times higher activity than alogliptin). Enzyme inhibition for compound A is seen to be lower than all B, C, D, and E because of the presence of electron-withdrawing chlorine atoms; for the rest of the compounds, IC_50_ is given in table [60] (Table 2) (Figure 10F).

### 5.3. Pyrazolopyrimidine Moiety

**Figure 11:**
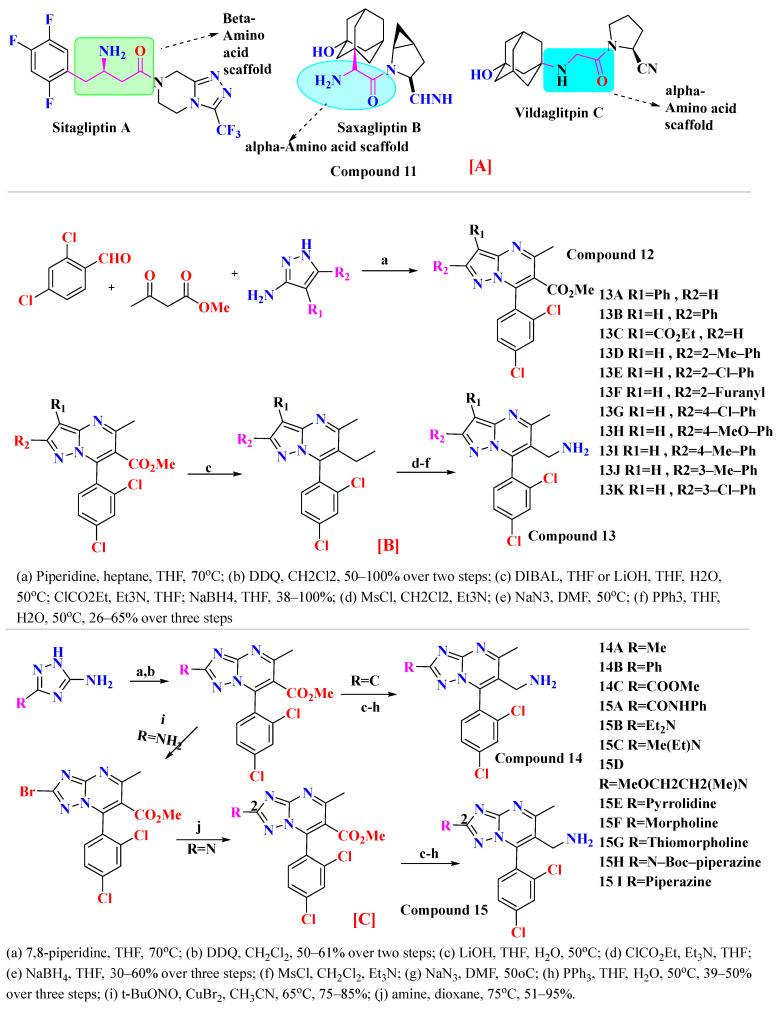
Structure of DPP-4 inhibitors: (**A**) Structures of selected DPP-4 inhibitors. (**B**) Compounds (**12**) and (**13**). (**C**) Compound (**14**) and (**15**).

Compounds **2** and **3** belong to a class of potent α-amino acid scaffold-derived drugs, which are dipeptide mimics, whereas 1 belongs to β-amino acid-derived compounds. Discovery of the pyrazolopyrimidine class possesses basic amine functionality that bound to the rigid system, which is a motif sharing commonalities with other non-amino acid-derived compounds (DPP-4 inhibitors), e.g., linagliptin and dutogliptin (Figure 11A). SAR of pyrazolopyrimidine states that several substituents on pyrazole moiety include aryl, carboxylic ester, alkyl, and substituted aryl are being used widely to explore in substitution region. All designed and synthesized substituent inhibitors were used in vitro against purified human DPP-4. Ample optimization could be done around the pyrazole scaffold; designed compounds showed fascinating potency against DPP-4; moreover, substitution at the R_2_ position in place of R_1_ showed >6-fold greater potency (1 = 141 nM and 2 = 22 nM). Establishing that, the remaining derivatives (4–11) were used to investigate substituting patterns on the phenyl group at the R_2_ position of the pyrazole scaffold. Substituting the phenyl group at the R_2_ position of the pyrazole ring illustrates that para-substitution is much more favorable than meta [59] (Figure 11B). Examining fused heterobicyclic systems directed us to optimize triazolopyrimidines as potent DPP-4 inhibitors excluding 32 and 40. Triazolopyrimidine derivatives were designed using two methods (i) one-pot reaction and (ii) Sandmeyer reaction [in which bromide can easily replace by nucleophilic amine generating amine]. Synthesized derivatives derived from pyrazolopyrimidines show significant diversity on fused 5-membered heterocyclic & further retained the potency of compounds. Most active compounds are founded to be which bears straight chain or cyclic secondary amines that holds whether Sulphur or oxygen (35=18 nM, 37=31 nM, and 38 = 29 nM), the substitution of triazole seen to be diminishing the potency to a lesser rate [29, 30, 33, 34 and 36 (50–73 nM)]. Furthermore, DPP-4 activity is seen to be extinct when substituting with secondary cyclic amine comprising nitrogen or carbo alkoxy group (31 = 137 nM, 39 = 442 nM, and 40 = 106 nM), observation tells us that substituting with pyrazolopyrimidine shows 2–3 folds more activity in comparison with triazolopyrimidine [59] (Figure 11C). 

### 5.4. Tetrahydro-Pyridopyrimidine Moiety

**Figure 12:**
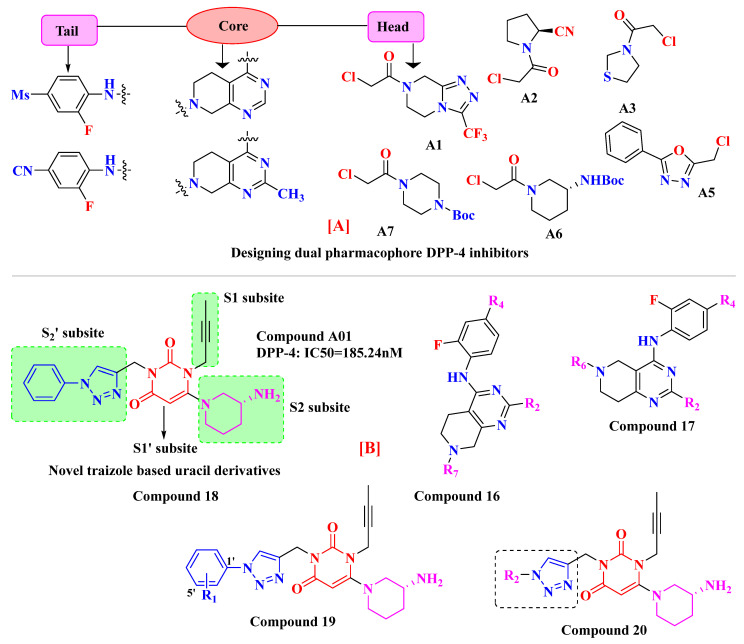
Structure of DPP-4 inhibitors: (**A**) Designing dual pharmacophore DPP-4 inhibitors. (**B**) Design of triazole-based uracil compound derivatives (Compound **16**–**20**) and linagliptin and alogliptin.

The wide diversity of gliptins scaffolds is being used in developing new DPP-4 inhibitors, although they mostly bind with the catalytic site of DPP-4 protease. Hydrophobic scaffolds, for instance, the 2-cyanopyrrolidine moiety of vildagliptin, trifluoro benzene, and thiazolidine of teneligliptin bind to S_1_ pocket (catalytic Ser630), whereas lesser hydrophobic moieties like tetrahydro-triazolopyrazine of sitagliptin, 3-aminopiperidine of sitagliptin seen to bind with S_2_ pocket of DPP-4. These mentioned fragments could play a role in designing DPP-4 inhibitors and serve as pharmacophores. It is observed in the study that designing a dual fragment modulator could target multiple receptors using combining dual pharmacophore mixture; this experimental study strategy was to retain pyrimidine as core (head) and aniline as substitution (tail) [61] (Figure 12A). Among all fragments used to develop DPP-4 inhibitors, A2 fragments displayed the best inhibition rate of 83.2% in inhibiting DPP-4; furthermore, A5 and A6 fragments showed lesser inhibition in comparison with A2. Substitution at position C2 with methyl leads to yield a much more potent compound; moreover, enhanced results were observed in 4,7-disubstituted tetrahydro-pyrido [4,3-d] pyrimidine nucleus. Compounds consisting of A2, A6, and A7 were found to be retaining potent DPP-4 inhibitory effects. Compound (**17B**) is identified to be the most potent DPP-4 inhibitor with an 83.2% inhibition at 10 micromolar (µM). The 2-cyanopyrrolidine scaffold of compound (**18C**) similarly occupies hydrophobic pocket S1 as vildagliptin whereas the nitrile group binds with Tyr547 and Tyr666 via forming H-bond. Tetrahydro-pyrido[4,3-d] pyrimidine scaffold tends to be seen interacting with Arg125 and Glu205 in S_2_ pocket; moreover, 2-fluoro-4cyanoaniline (tail fragment) interacts with Phe357 present in S_2_’extensive subsite, and cyano group interacts via forming H-bond with Arg669 [61] (Table 3) (Figure 12A).

### 5.5. Triazole-Based Uracil Moiety

Choosing a uracil-based configuration for making possible interaction with S_1_’ subsite, whereas adding a 2-butenyl group at the N-3 position of uracil leads to the formation of a bond with the S_1_ subsite of DPP-4 protease. Adding 3-amino piperidine at the C-6 position of uracil was seen to be forming a salt bridge interaction with Glu206 and Glu205 (S_2_ subsite). Furthermore, attaching the 1,2,3-triazole ring at the N-1 position of uracil moiety tends to bind with S_2_’ subsite because of two causes (i) substitution at position-1 of 1,2,3-triazole moiety could be easily changed for SAR (structural activity relationship) studies (ii) 1,2,3-triazole might interact with Trp629 via π-π interaction likewise as quinazoline moiety of linagliptin. Compound (**19A**) showed predicted interactions as mentioned above, but the potency of (**19A**) against DPP-4 was found to be lesser than expected when compared with alogliptin and linagliptin. Designing derivatives of (**19A**) by substituting various groups at the N-1 position of triazole moiety yields several compounds with a variation in potency [62] (Figure 12B). Compounds with intriguing inhibition rates at 100 nM were further selected for their IC_50_ values evaluation; analogues synthesized and mentioned in the below table showed the best inhibition rate and were further analyzed for IC_50_ value calculation. The initial compound (**19A**) was used as starting point with an IC_50_ of 185.24 nM and for SAR studies too, focusing on phenyl ring attached to 1,2,3-triazole [(from N-1 position) compounds from **19A**–**F**)]. Substitution of fluoro atom at ortho or para position of benzene seen to be enhancing the potency of the compounds, noticed that adding fluoro on para position tends to increase the potency of (**19B**) by 3-fold whereas for (**19C**) exhibited 135.45 nM. Introducing two floro atoms on benzene reduced the potency with IC_50_ of 243.67 nM; adding chloro atom on the benzene group (at meta-position) displayed potential against inhibiting DPP-4 but replacing chloro atom from meta-position to other para or ortho led to decrease the potency of the compounds. Adding the methoxy group at the meta-position exhibited good potency against DPP-4 but lesser than marketed drugs (alogliptin and linagliptin) in comparison [62]. Different carbon chain lengths of aliphatic carboxylic acids were added at position-1 of triazole moiety which further led to an increase in DPP-4 inhibitory activity. Analogs holding propanoic acid & acetic acid revealed improvement in the potency of about 2–3 folds in comparison with the (**19A**) compound, (**20F**) analog showed [consist of (E)-but-2-enoic acid] foremost potency with bearing an IC_50_ of 9.56 nM as nearby as of alogliptin. These conclusions indicate that the addition of a C-chain in the carboxylic acid group (R_2_) could lead to the synthesis of a potent compound with intriguing DPP-4 inhibitor activity [62] (Figure 12B) (Table 4).

### 5.6. Uracil-Based Benzoic Acid and Ester Derivatives

**Figure 13:**
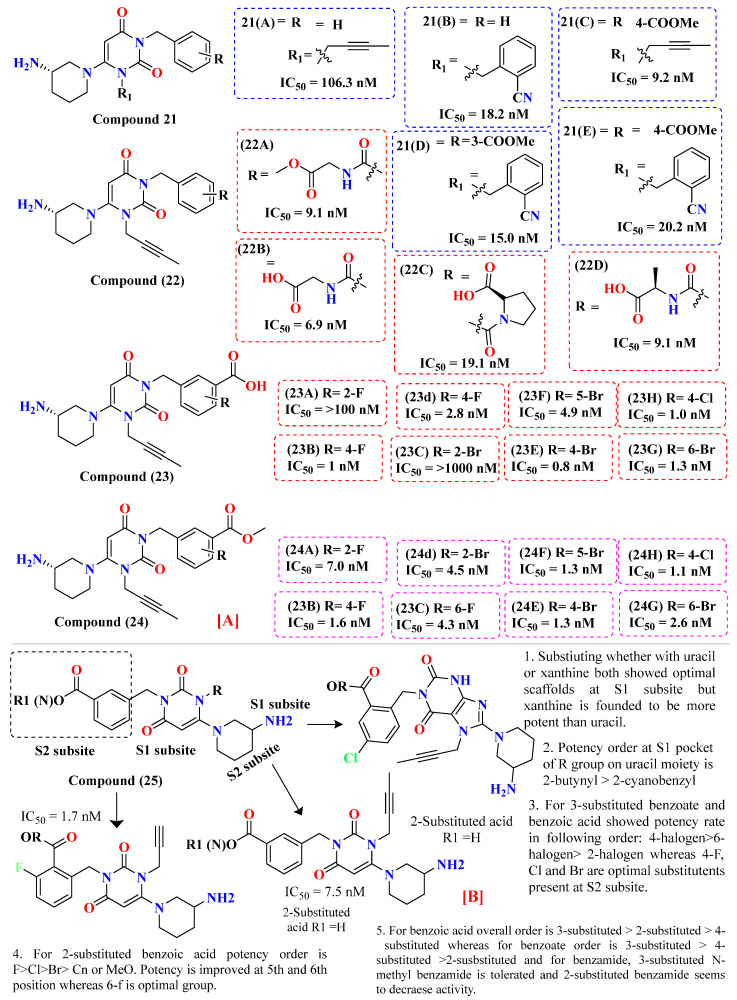
Structure of DPP-4 inhibitors: (**A**) Potency of synthesized derivatives (**21**–**24**) against DPP-4 protease with IC_50_. (**B**) SAR compact of benzoate and benzoic acid and its analogs-based DPP-4 inhibitor (**25**).

Li et al. developed and synthesized compound 3 derivatives using carboxyl scaffold moiety compounds with much more potency of about 15 folds when compared with 7 (without carboxyl moiety) compounds. Compounds holding esters of carboxyl derivatives exhibited lower DPP-4 inhibition with albeit enhancement in oral absorption, on optimization of substituting of 2-substituted benzoic acid with 3-substitution leads to the discovery of compound observing that substituting ester could be a promising lead us to the potentially active compound. Compound (**24B**) exhibits an optimal compound showing better pharmacokinetic studies and excellent metabolic studies. The above results indicate that the carboxyl group binds with different positions of residues playing a vital role in each interaction with residues. Synthesized compound (**21A**) (without carboxyl group) acted as a potent DPP-4 inhibitor with an IC_50_ of 18.2 nM; however, compounds holding esters **21A**–**E** seem to be retaining inhibitory activities, among which (**21D**) were found to hold ester at 3′-position with an IC_50_ of 15 nM whereas, (**21E**) with IC_50_ of 20.2 nM holding ester at 4′-position but **21C** seems to be lost the inhibition activity and the best compounds amongst their class in regard showing similar inhibition potency, collectively both 3-substituted ester & benzoic acid coupled with 2-butnyl were best potent compounds & they selected for further evaluation of SAR. Further, all the focus was centered on the conjugation of several amino residues and their esters (**22A**–**D**) to the carboxyl group. On conjugation of glycine & ester produced compounds **22A** and **22B** with identical inhibition potency, however introducing proline in both conformations, whether R or S displayed lower potency of compounds with an IC_50_ of 19.1 & 34.6 nM, whereas their esters led to a reduction in DPP-4 inhibition. As mentioned above benzene ring holding a halogen group could be beneficial for increasing DPP-4 inhibition; hence the addition of halogen on benzene scaffold (3-substituted benzoic acid) and (3-substituted ester) is introduced to enhance DPP-4 potency. Introducing fluoro group at 2,4,6-position of the compound as it is mainly demotic bioisosteric substitution for H-atom with a potency order of 4-f > 6-f > 2-F among which **23B** compound holding F atom at 4-position indicated most potent within their class with an IC_50_ of 1.0 nM whereas **23D** showed IC_50_ of 2.8 nM. After studying the SAR of fluorine, replacing fluorine with bromine at 2,4,5 & 6-position highlighted a slight increase in DPP-4 inhibition potency compared with fluoro-based compound derivatives, specifically at 4,6-position. Substitution with bromine on **23E** displayed IC_50_ of 0.8 nM, i.e., five-fold more potent than alogliptin. Moreover, **23C** and **23A** lost most of their potency against DPP-4, yielding an IC_50_ of >1000 nM [63]. Introducing halogen on benzene moiety of the ester, all ester-derived derivatives synthesized for class 24 demonstrated higher DPP-4 inhibition potency with IC_50_ < 7 nM. Fluorine based-derivatives (**24A**,**B** and **24D**) showed an IC_50_ of 7, 1.6, 4.3 nM with potency order of 4-F > 6-F > 2-F, 4-bromo substituted derivatives (**24C**–**G**) slightly low digit nM inhibition with IC_50_ values of 4.5, 1.3, 1.3, 2.6 nM whereas, 24h highlighted a little bit lower single-digit nanomolar DPP-4 inhibition [63] (Figure 13B). To infer inhibition potency of synthesized compounds, docking was performed for ester and benzoic acid based-compounds (**23** and **24**). Mentioned compounds founded to be preserving two common binding sites as 2-substituted acid, the primary amine of 3R-aminopiperidine binds with Glu205 & Glu206 via salt bridge interaction, whereas the 2-butenyl group gets accommodated at the S_1_ pocket to the form hydrophobic interaction. Furthermore, some vital changes were evaluated at S_1_& S_2_ subsites. The Carboxyl group present at 3-substituted benzoic acid impacts interaction as well as the orientation of selected uracil and benzoic acid scaffold. The Carboxyl group of compoundsformed a salt bridge with Lys554 primary amine, whereas the uracil scaffold compound interacts via π-π stacking. Compounds with 3-substituted benzoic acids of **23B**, **23E**, and **23H** leaned towards amino nitrogen residues of Trp629 & Lys554 at S_2_’ subsite; all these H-interactions are essential for DPP-4 inhibition. Ester presents in 24B finds to occupy the same binding mode as 18b, 18e, and 18h, 2 oxygen (ester) present in compound engaging in 2 key H-bond with Trp629 & Lys554 [63] (Figure 13A).

### 5.7. Quinoxaline Scaffold

**Figure 14:**
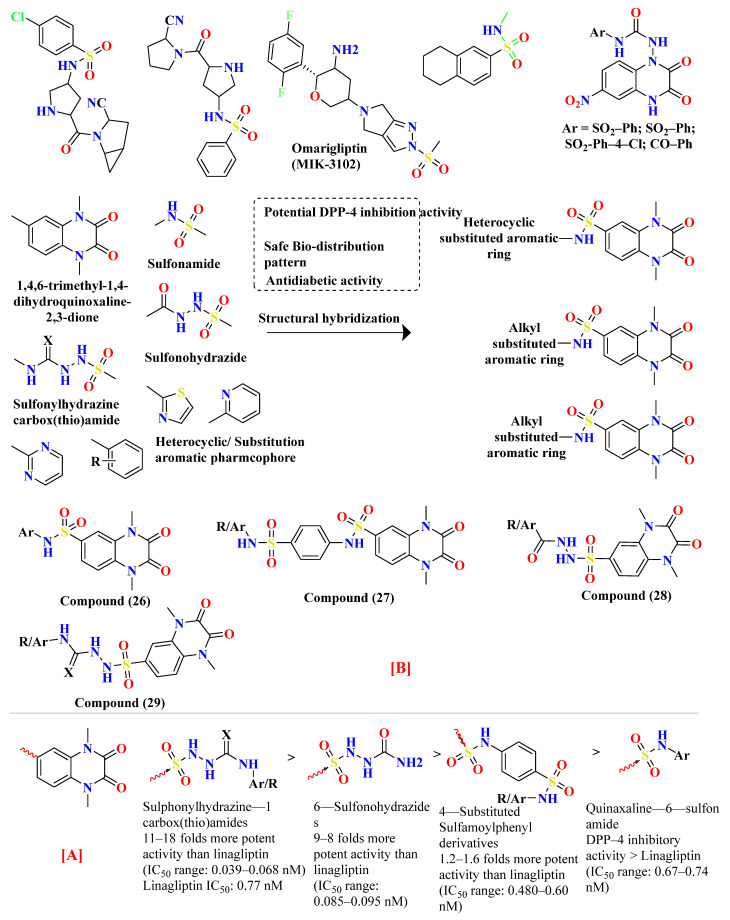
Structure of DPP-4 inhibitors: (**A**) SAR evaluation on basis of data of DPP-4 inhibition classification. (**B**) Scheme strategy for developing sulfonamide-quinoxaline based compounds.

Various substitutes **26A**–**26F** conjugation were produced using 2,3-dioxo-1,2,3,4-tetrahydroquinoxaline with various aromatic moieties via 6-sulfonyl as linker exhibit-ing excellent DPP-4 inhibition activity with IC50 value varying from 0.67–1.28 nM. However, **26A** tends to lose the DPP-4 inhibition. Suppression in DPP-4 inhibition activity occurred due to substituting the thiazolyl group in the **26A** compound, further seen to lose the entire potency towards DPP-IV. 4-substituted sulfomyl-phenyl, pyrim-idinyl & pyridinyl derivatives **27A**, **27C**, and **27D** enhanced potency up to 1.2–1.6 times than the data compared with linagliptin (standard drug). Conjugation of 2,3-dioxo-1,2,3,4-tetrahydro quinoxaline-6-sulfonamide with 4-substituted sulfomylphenyl motif as a linker in synthesizing molecule **27** displayed a vital increase in the inhibitory state. A slight variation in the potency of the compound is observed after the addition of thi-azolyl derivative, yielding an IC50 value of 0.93 nM, whereas the enhancement in inhib-itory potency could be because of the additional presence of sulfamoyl derivative. Further prolongation of 6-substituted sulfonohydrazide derivative to sulfonyl-N-substituted hydrazine-1-carbox(thio)amide, developing several corresponding derivatives of **29** with IC50 values ranging from 0.039–0.068 nM and binding within an active pocket of DPP-4 protease with an additional interaction which tend to increase the potency of the synthesized derivatives **29A**, **29B**, **29G**, **29F** of specified class however, **29D** and **29H** ascertained to lost their inhibition potency against DPP-4 holding p-chlorophenyl and p-methoxyphenyl derivatives. On the other hand, elongating the 6-sulfonamide linker to 6-sulfonohydrazide leads to the pretense of intriguing inhibitory activity of ligand derivatives **28A**–**C**. Molecules **28A**–**C** showed restraining in DPP-4 protease with an IC50 of 0.085–0.095 nM with an increase in potency of 8–9 folds higher when compared with linagliptin. Compounds **28A**–**C** detected potency could be explained because of the existence of O & N atoms forming H-bond interaction with active pocket amino residues. **29G**, **29A**, **28A**, and **27D** showed the best potency in sup-pressing DPP-4 activity & also chosen for further selectivity assays [63] (Figure 14A).

Design for development of new compounds (**26**–**29**) using synthetic approaches possessing 1,4-dimethyl-2,3-dioxo-1,2,3,4-tetrahydro quinoxaline-6-sulfonamide moiety. Most active synthesized compounds **28A**, **29A**, **29F**, and **29G** founded to be useful in suppressing DPP-4 activity by binding the target molecules in the active pocket of DPP-4 protease (Figure 14A). Wide spectrum of scaffolds is being used in developing DPP-4 inhibitors with efficient biological activities; sulphonamide moiety holds an impelling pharmacophore in field of drug discovery & medicinal chemistry. Widely used sulfonyl moiety has two functionality (i) binds with active pocket amino residues interacting via H-bond, (ii) incorporates structural core nucleus & constraints side chain, permitting them to fit in active pocket binding sites. Mentioned above are sulfonyl-based compounds and their analogs; for instance: I&II yield an IC_50_ of 6.7 & 39 nM. A series of compounds were synthesized holding 1,4-dimethyl-2,3-dioxo-1,2,3,4- tetrahydro quinoxaline scaffold conjugating with alkyl substituted aromatic ring and heterocyclic rings at 6-position via using linkers like sulfonohydrazide, sulfonamide, and sulfonyl hydrazine-carboxy(thio)amide to develop potential DPP-4 inhibitors resulting in anti-diabetic effect [64] (Figure 14B), (Table 5).

### 5.8. Glycinamide, Glycolamide and β-Amino Carbonyl 1,2,4-Triazole Scaffold

**Figure 15:**
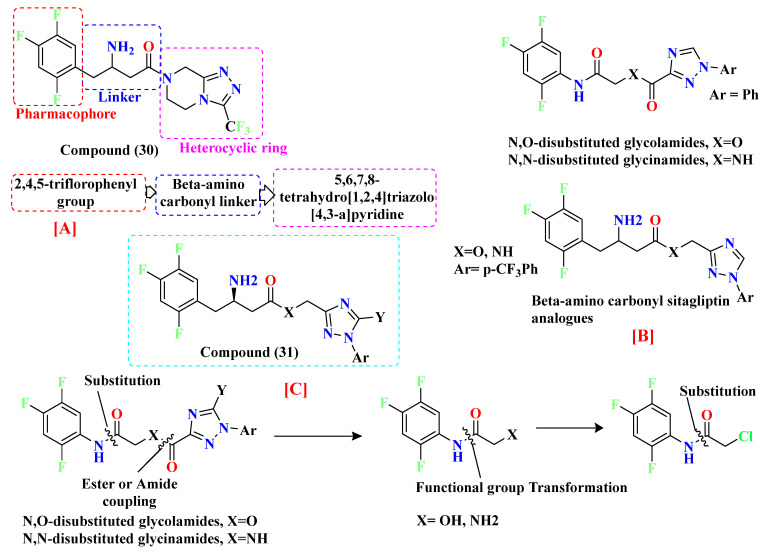
Structure of DPP-4 inhibitors: (**A**) Different fragments used in developing sitagliptin. (**B**,**C**) Newly developed molecules consisting of N, O-disubstituted glycosamides 3, N, N-disubstituted glycinamides 4, and β-amino carbonyl 1,2,4-triazoles.

Sitagliptin is fragmented into three parts: β-amino carbonyl (acts as a linker between two scaffolds), 2,4,5-triflorophenyl (as nucleus pharmacophore), and 5,6,7,8-tetrahydro-[1,2,4]triazolo [4,3-a]pyridine fragment (Figure 15A). In vitro evaluation of developed compounds and SAR of N, O-disubstituted glycolamides 3, N, N-disubstituted glycinamides 4 and β-amino carbonyl 1,2,4-triazoles 5&6. Evaluation of glycolamides and glycinamides with 1,3-disubstituted 1,2,4-triazole scaffolds were analyzed for evaluating in vitro studies against DPP-4 protease (Figure 15B,C). For evaluating the DPP-4 inhibition activity of compounds by introducing β-amino carbonyl 1,3-disubstituted 1,2,4-triazoles in compounds, **31A** and **31B** found efficient as inhibition properties. Moreover, adding β-amino amide in the **31B** compound displayed high vital inhibitory efficacy against DPP-4 protease with an IC_50_ of 34.4 nM whereas the **31A** compound showed no major significance and an IC_50_ of the compound remained at 775 nM. Numerous derivatives were synthesized using two linker chains (i) β-amino amides (**31D**, **31F**, **31J**) and (ii) β-amino esters (**31C**, **31E**, **31G**, **31I**, **31K**) also modifying 1,3,5-trisubstituted 1,2,4-triazole and most of the compounds showed moderate DPP-4 inhibitory activity, values ranging from 34.4 to 497 nM. Analogs like **31B**–**31H** showed the best inhibition among synthesized compounds [65] (Figure 15C). Compounds were docked on the same site as sitagliptin and showed three hydrogen bonds binding with Glu205, Glu206, and Tyr662 residues and two π-π interaction with Phe357 and Tyr662. When **31E**, **31B**, and **31J** compounds were compared with sitagliptin showed identical interaction with DPP-4 confirming 3-H bonds with residues Glu206, Glu205, and Tyr662, two π–π interaction with Tyr662 and Phe357. Compound **31B** achieved a docking score of -150 kcal/mol because of the presence of the 4-(trifluoromethyl) phenyl group, which displayed it interacting with Arg358 residue via Van der Waal interaction. On the basis of docking analysis, it is established that 1,3,5-trisubstituted 1,2,4-triazole compound **31E** and **31J** showed better docking results due to existing of an additional aromatic ring on triazole moiety, which also furnishes strong π–π interaction with Phe357 amino acid than **31B** (Figure 15C) (Table 6).

### 5.9. Comparing Adamantane and Fluorophenyl-Based Scaffold Molecules

#### 5.9.1. Saxagliptin and Vildagliptin

**Figure 16:**
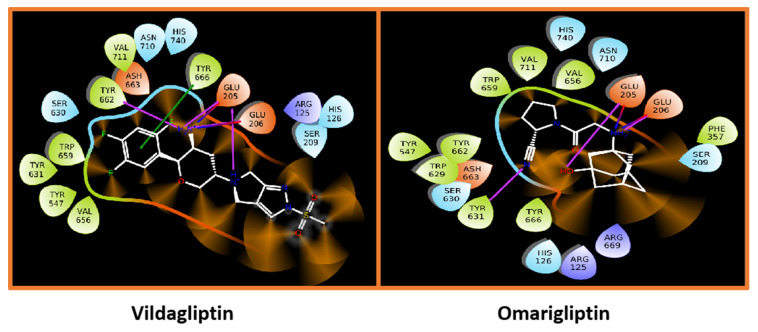
2D diagram of vildagliptin (PDBID:6B1E) and omariligliptin (PDBID:4PNZ).

The discovery of amantadine as a potent inhibitor for influenza A infection and anti-Parkinson was made in 1960. Moreover, further studies on adamantine scaffold lead to the discovery of more potent antiviral drugs like tromantadine and rimantadine. Several derivatives showed marked potent inhibitory activity against human immunodeficiency viruses (HIV), and decades after researchers studied adamantine scaffold to treat T2DM [66]. As both drugs (vildagliptin and saxagliptin) are designed as peptide mimetics, when observed, they overlap with the P_1_ and P_2_ residue of substrate peptide. Cyanopyrrolidine moiety interacts via the S_1_ subunit, whereas the hydroxy group of the adamantyl ring binds to the S_2_ subsite, forming a covalent bond between the Ser630 (catalytic traid) & nitrile group of compounds. The reason saxagliptin for being five times more potent than vildagliptin is the presence of cyclopropanated over cyanopyrrolidine ring of saxagliptin despite the reason for introducing cyclopropanated was to enhance chemical stability to the compound by providing additional hydrophobic interaction with the residue of S_1_ subsite furthermore forming a direct H-bond with OH group of saxagliptin could contribute to enhancing its potency [29] (Figure 16) (Table 7).

#### 5.9.2. Sitagliptin and Omariligliptin

**Figure 17:**
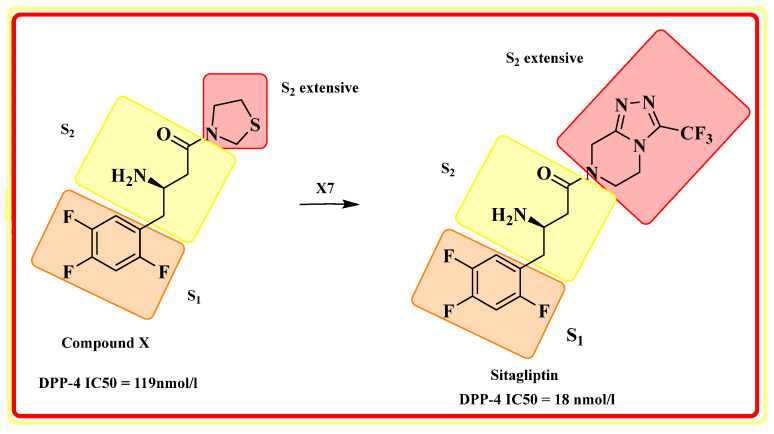
Effect of compounds binding with S_2_ extensive subsite illustrated by compound X and sitagliptin IC_50_.

**Figure 18:**
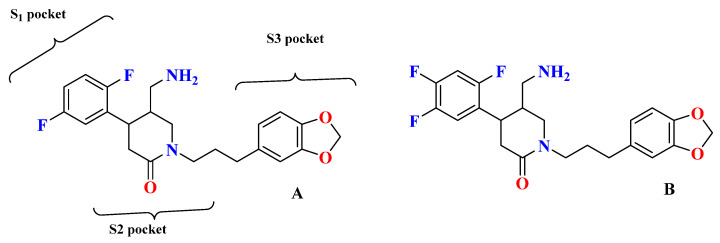
Synthesis of fluorophenyl-piperidine-based DPP-4 inhibitors.

Tri-fluorophenyl moiety of sitagliptin binds to the S_1_ subsite, whereas tri-fluorophenyl forms interaction with S_2_ extensive subsite leading to cause a tremendous yield with a good IC_50._ Favorable binding affinity could be because of 2 reasons, (i) hydrophobic interaction because of tri-fluorophenyl scaffold and (ii) interaction with S_2_ extensive subsite promotes activity by 7-fold. It is revealed in the study that the higher the contact area of the ligand with protein more tightly it will bind with the S_2_ extensive subsite concluding stronger hydrophobic interactions mediated by the “anchor lock domain,” which further relates to the residence time of DPP-4 inhibitor resulting in longer in vivo duration of action [29] (Figure 16 and Figure 17) (Table 8). Kumar et al. docking studies of A compound were d using the extra precision (XP) glide docking method confirm that it occupies all three binding pockets (S_1_, S_2,_ and S_3_), showing intriguing interaction via forming H-bond with residues (Glu206 and Glu205) (Figure 18). Adding an amino-methyl group in the piperidone ring is thought to improve the pharmacodynamic and pharmacokinetic profile of the drugs; compound **1** is founded to have 79.5% of bioavailability with potent activity.

### 5.10. DPP-4 Inhibitors under Pipeline

Since the discovery of sitagliptin (first DPP-4 inhibitor), about 10 of gliptins are under clinical progress and some of them have been commercialized each holding unique variability such as pharmacokinetic, pharmacodynamic and selectivity against DPP-4. GLP-1 and GIP both incretins help in preventing getting degraded via DPP-4 and increase insulin biosynthesis and regulates in glycemic level. However, there is keen interest founded in development of novel DPP-4 inhibitor as some of the marketed drugs have adverse effects such as gastrointestinal problems, skin reactions and majorly high risk of developing pancreatitis have been observed experiencing severe pain in upper abdomen.

### 5.11. Challenges in Development of DPP-4 Inhibitors

DPP-4 inhibitors belong to oral class of antidiabetic drugs that facilitates glycemic control reducing hypoglycemic control and weight control in patients. Monotherapy or di or tri combination therapy (for instance metformin and thaizolidinedione) is being preferred in treatment. Off target prohibition of DPP-4 inhibitors could lead to several toxicities such as skin reactions, immune dysfuction and impaired healing [68].

#### 5.11.1. Acute Pancreatitis

FDA warned on insertion of drugs directly acting on incretins, several cases of pancreatitis were observed in postmarketing analysis but it should be noted that patients with hyperglycemia and T2DM are risked of pancreatitis [68].

#### 5.11.2. Cardiovascular Risks

**Figure 19:**
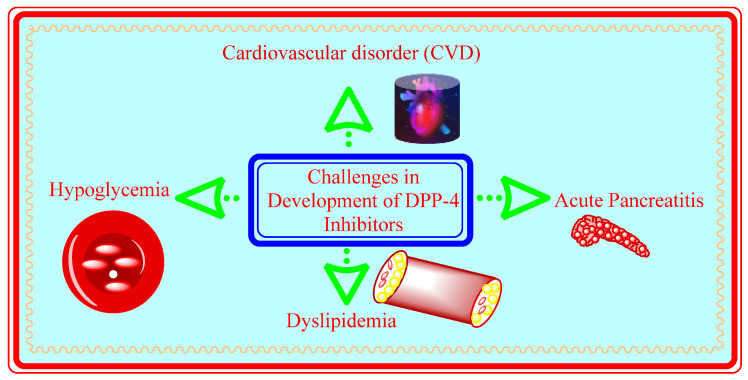
Illustrating challenges during development of DPP-4 inhibitors.

Intensive control could associate higher cardiovascular risk (especially linagliptin). Using animal model studies, GLP-1 activation is related with limiting size of area of Myocardial infarction [68]. DPP-4 is founded in almost every cell/tissue and express exopeptidase activity and numerous other vascular function, cell survival and homing with inflammation. DPP-4 plasma activity helps in correlating cardiac dysfuction in experimental and humans models of HF signaling direct relation between CV and DPP-4 inhibitors (Figure 19) [69].

### 5.12. Current Scenario

Numerous marketed gliptins are being used as DPP-4 inhibitors (as monotherapy or as well as in combination). These all drugs falls under competitive inhibitors and founded to be responsible for about 70% of inhibition of plasma DPP-4 with some reported side effects such as nasopharyngitis, respiratory tract infections, headache and major setback is founded to be pancreatitis and hypersensitive reactions [68].

In respect for most of the compounds it shows low protein binding in plasma 10% for vildagliptin, negligible for saxagliptin and 38% for sitagliptin as per their pharmacodynamic, excretion and pharmacokinetic properties. Linagliptin finds to bind with plasma proteins extensively & at the therapeutic dose, i.e., 5 mg primarily all the DPP-4 inhibitors will be protein bounded. Higher the protein binding lesser be the drug excretion via glomerular filtration that also makes linagliptin whose excretion in urine is founded to be <6%. Since DPP-8/DPP-9, DPP-10, FAP all falls under DPP-4 family proteases, to minimize off-target side effects of inhibitors deliberately used as therapeutically to have selective enzyme specificity. At present sitagliptin is available as single drug/agent but with a 50 mg of daily dosage association produces 80% inhibition of DPP-4 activity and founded to be extreme selective (upto 2600 times). Moreover, sitagliptin helps in improving surrogate β-cells markers function in humans as well as animal studies. Saxagliptin is about 10 times much more effective in comparison with sitagliptin and vildagliptin. Incorporating cyanopyrrolidine moeity and adamantane increases its β-cells function when imployed as monotherapy or in combination form. FDA also endorsed saxagliptin use in improving glycaemic control in curing type-2 diabetes mellitus [68].

Vildagliptin is much similar drug when compared with saxagliptin, 100 mg of vildagliptin can supress DPP-4 activity completely and also helps in stimulating cell mass (as per in vivo studies). Despite of structural heterogeneity which possess pharmacokinetic properties for instance, linagliptin is xanthine based moiety structure and have half life of 184 h much longer than sitagliptin. Handling patients with renal dysfunction, differences in drug metabolism path could also be pivotal. As linagliptin binds strongly with DPP-4 it quickly becomes saturated at lower doses hence, free linagliptin in blood stream gets rapidly eliminated, however it gets excreted via kidney by breaking down the drug into active metabolite (with 85% bioavailability) [70]. Five gliptins (saxagliptin, sitagliptin, alogliptin, vildagliptin and linagliptin) falls under small molecules with identical clinical safety, profile and efficacy. Various studies illustrates comparison of DPP-4 inhibitors. Major difference of marketed gliptins includes: target selectivity, long or short half life, potency, bioavalability and binding efficiency to plasma proteins, excretion route, metabolism and dosage adjustment to minimize side effects such as pancreatitis, liver & renal insufficiency as well as potential drug-drug interactions. On average, gliptins are tend to show glycated hemoglobin (HbA1c) drop of 0.5–0.8% for about 40% diabetic subject targets for a goal of <7% HbA1c level [71].

## 6. Analysis of Several DPP-4 Inhibitors

DPP-4 protease is made up of three domains, amongst which 8-stranded β-propeller present at N-terminal and serine protease at C-terminal. The active binding pocket of DPP-4 protease is located at the N-terminal of serine protease. It consists of catalytic triad His740, Ser630, and Asp708, which occupy a cave-like projection belted by hydrophobic residues. S_1_ and S_2_ pockets comprise protein around the active site; the S_1_ pocket is lined by several amino acid residues comprised of Ser630, Tyr547, Trp659, Tyr631, Asn710, Tyr662, Val656, Tyr666, and Val711 residues which are also the reason of hydrophobic environment of S1 pocket whereas, S_2_ pocket comprises of Arg125, Glu205, Glu206, and Pro550 residues. Asp708, Trp627, Lys544, Arg358, Val207, Ser209, Phe357 residues all together consist of S_2_ extensive subsite which encircle S_2_ pocket of DPP-4. Site mapping highlights the significant role of residues Tyr547, Trp629, and Lys554 are high-rank active site binding pockets of protease-producing enzyme-inhibitor complexes. Trp629 residue forms a catalytic pocket in DPP-4 protease, whereas Tyr547 stabilizes oxyanion in the DPP-4 catalytic site. S_1_ and S_2_ subsites are mandatory against DPP-4 selectivity, but adding a pharmacophore capable of forming interaction with Lys544 residue enhances DPP-4 inhibition activity [59].

**Figure 20:**
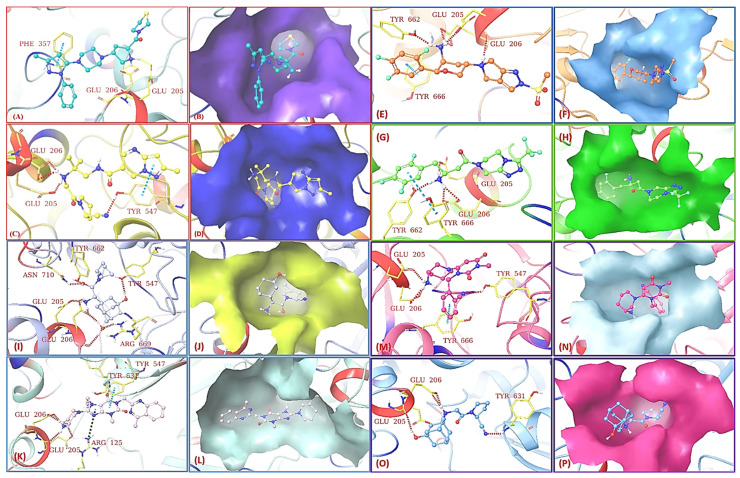
Docked pose of Teneligliptin (PDBID:3VJK) (**A**) 3D interaction, (**B**) Surface view) and Anagliptin (PDBID:3WQH) (**C**) 3D interaction, (**D**) Surface view) showing various kinds of bond forming with amino acids in binding sites of DPP-4 with 3D interaction. Docked pose of Omarigliptin (PDBID:4PNZ) (**E**) 3D interaction, (**F**) Surface view) and sitagliptin (PDBID:1X70) (**G**) 3D interaction, (**H**) Surface view) showing various bond formation with amino acids in binding sites of DPP-4. Docked pose of Saxagliptin (PDBID:3BJM) [displayed in 3D interaction (**I**) 3D interaction, (**J**) Surface view)] and Linagliptin (PDBID: 2RGU) [displayed in (**K**) 3D interaction, (**L**)Surface view] compounds showing various bond formation with amino acids in binding sites of DPP-4 with 3-D interaction. Docked pose of Alogliptin ((**M**) 3D interaction, (**N**) Surface view) and Vildagliptin (PDBID:6B1E) ((**O**) 3D interaction, (**P**) Surface view) compounds showing various kinds of bond forming with amino acids in binding sites of DPP-4 with 3D interaction. Figure (**G**), alogliptin (PDBID:2ONC) highlights the H-bond formation of Glu205 and Glu206 residue with the 3-aminopyridine ring, Tyr547 forms H-bond with the benzonitrile ring of the compound, whereas Tyr666 was founded to be forming π-bond with the same ring (benzonitrile). In the graphical picture H, it is observed that all of the residues (Glu205, Glu206, and Tyr631) interact via H-bond with the compound (vildagliptin).

**Figure 21:**
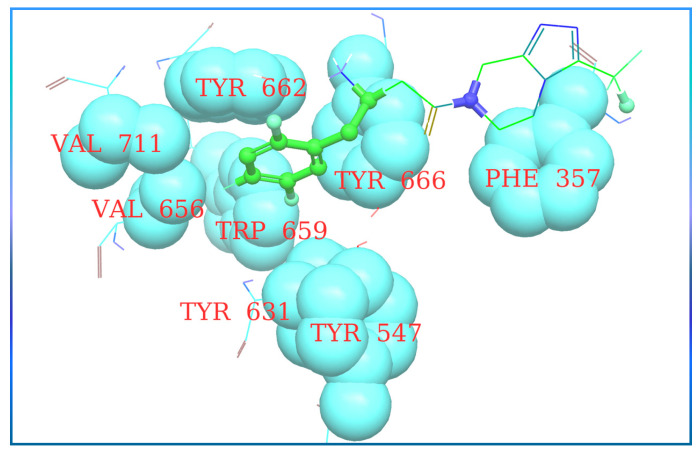
XP visualization pose of Sitagliptin showing hydrophobic enclosure (PDBID:1X70).

The phenyl-pyrazole moiety of teneligliptin gets accommodated at S_2_ extensive position with Phe357 whereas the pyrrolidine moiety perfectly fits in S_2_ extensive pocket binding hydrophobic interaction with Arg358, Ser209, and Val207 residues. The pyrrolidine ring of teneligliptin interacts with Glu205 & GLu206 holding perfectly in the S_1_ pocket. Docking of crystal DPP-4 (PDBID:3VJK) against teneligliptin (pyrrolidine-based inhibitor) forms H-bond with Glu205 and Glu206 as earlier seen in Figure 20A,B); moreover, it is observed that pyrazole ring of the compound is interacting via forming π-bond with phenylalanine residue (PHE357). Figure 20A represents the formation of a 3-H bond and one π-interaction of teneligliptin. The molecule’s pyrazole ring forms one π-interaction with Phe357 residue (N…C). Pyrrolidine ring forms, and pyrazole moiety of teneligliptin interacts strongly with their site-specific residues, forming 3-H bonds with pyrrolidine and one π-bond with the pyrazole scaffold of the compound. The surface view of the protein with teneligliptin in complex form is displayed in Figure 20B. Docking of crystallographic anagliptin & refinement data is displayed in Figure 20C (PDBID: 3WQH). Displayed complex structure of DPP-4 protein with anagliptin consisting of dimeric protein in an asymmetric unit, single anagliptin bonded with each protein residue. Overall, anagliptin was revealed to have three interactions with DPP-4 (i) Cyano group forms π-interaction at S_1_ subsite with Tyr547 (proton donor), (ii) Second interaction seen to be at S_2_ subsite with secondary amines forming salt bridges with Glu205 and Glu206 residues (iii) carbonyl group present at pyrazolopyrimidine moiety underwent forming H-bond interaction with Tyr547 indicating 3rd subsite is S_2_ extensive. As displayed in Figure 20C above, interactive subsites of anaglitpin are S_1_, S_2,_ and S_2_ extensive subsites. The Cyano group was seen to have dipole interaction with S_1,_ but it further didn’t transform into a covalent bond with the oxygen of Ser630. The pyrazolopyrimidine ring of the anagliptin forms an interaction with Tyr547 residue via π-interaction (N…C) & another H-bond seen to be forming with heterocyclic cyanopyrrolidine moiety of the compound via interacting with Tyr547 (NH…C). Three H-bonds take place, among which two are forming with Glu206 (NH…O and NH..OH) and one with Glu205 (NH…OH) residues; these interactions are seen to be forming with the N-atom present on α-amino acyl chain of the compound. The vital point observed in this docking pose is that the pyrazole and pyrrolidine (N..C=O) ring makes hydrogen and π-bond with the same amino acid, i.e., Tyr547. Interaction of anagliptin with S_2_ extensive subsite is seen to be contributing to enhancement to affinity as well as selectivity as teneligliptin does too [72] (Figure 20). In given Figure 20E, the interaction of omarigliptin with the S_2_ pocket of DPP-4 is shown. Omarigliptin and sitagliptin were noticed to be interacting in similar conformation with the same DPP-4 active site and also share identical key interactions. The essential amino group present on the tetrahedron ring is seen to be forming a salt bridge with Glu205 and Glu206 residues. Fused five-membered ring forms π-π stacks with an amino acid residue named Tyr666. The highlighted molecule is shown in a stick (thick brown) model indicating the formation of an H-bond with amino acids (Glu205 and Glu206) of a hydrophobic pocket (S_2_). The crystal structure of human DPP-4 (PDBID: 4PNZ) was used to dock against omarigliptin. Omarigliptin accommodates in S_1_ active subsite where it binds with several hydrophobic residues like Tyr666, Val711, and Tyr662. The nitrogen atom of the Pyrrolidine ring on omarigliptin forms an H-bond via interacting with the carbonyl group of Glu205, whereas the N-atom of morpholine moiety forms a 2-H bond with C=O (carbonyl group) of Glu206 amino acid. Moreover, the N-atom of the 2,5-diflorophenyl ring formed π-bond with Tyr666 (figure E) (Figure 20). Surface view of omarigliptin and sitagliptin is shown in Figure 20F,H illustrating the binding pocket of the protein and active binding sites of protein forming interaction with specific residues. Figure 20G depicts the DPP-4 interaction site disclosed (using PDBID: 1X70) & it seems like the β-amino butanoyl group of the molecule appears to be forming three interactions. The docking pose of sitagliptin shows that the amine group on the β-amino chain accommodated very well in the protein active site (Figure 21). Depicted interactions of sitagliptin with DPP-4 protein are retained in the class of inhibitors. The β-amino linker formed 3-H bonds with Glu205, Glu206, and Tyr662, respectively, and the compound’s hydrophobic moiety, i.e., trifluoro-phenyl forms an additional π-interaction with Tyr666 residue. The triazolopiperazine scaffold is stacked over side chain residues of Phe357, whereas several amino acid residues like Arg358 and Ser209 loosely surround trifluoro-phenyl moiety. Piperazine is facing side-to-face against Gluc205 whereas the trifluoro-phenyl scaffold is forming front-to-side face hydrophobic interaction with Tyr666, and the N-atom of amine group present on the β-chain linker describes main chain oxygen of Glu205.

Linagliptin was docked against mentioned DPP-4 protein (PDBID: 2RGU) which plays a crucial role in the pathogenesis of T2DM. Linagliptin and sitagliptin exist in similar conformation in a complex form, with DPP-4 residing in a similar manner forming interaction with similar residues. Conformation of the piperidine ring in linagliptin enhances binding affinity, whereas, in sitagliptin, piperidine moiety is missing. Various interactions of the molecule with protein are observed, as given in Figure 20E; linagliptin has shown inhibitory potential towards DPP-4. Glu205 and Glu206 were the most common amino acids found to be forming an H-bond with N-atom present on the piperidine ring, whereas carbonyl (C=O) of xanthine moiety was forming an H-bond with an amine group (NH_2)_ of Tyr631 present at S_1_’subiste of DPP-4. Adding to the statement, Tyr547 linked with the xanthine moiety of the ring via π-bond, whereas Arg125 was founded to be forming a π-cation bond with the same xanthine moiety. Evaluating molecular docking of saxagliptin with protein (PDBID:3BJM) was carried out. H-bond interaction was seen based on their inhibitory potential, residue interaction and H-bond is further described in Table 9 (Figure 20). In attached Figure 20F, dotted highlighted lines (H-bond) attract our attention presenting that all of the residues are forming H-bond with selected compound. The graphical picture of docking represents that Glu205 residue is interacting via forming a 3-H bond, among which two are formed with an N-group attached to an adamantane ring, whereas one of them is forming an H-bond with water. Moreover, saxagliptin interacts with hydrophobic DPP-4 residues forming H-bonds with Asn710, Tyr547, Glu205, Glu206, and Tyr662. Vildagliptin and saxagliptin hold the same scaffold with different conformations and slight variation at the cyanopyrrolidine ring of the molecules; another vital point while differentiating is vildagliptin holds methano-prolinenitrile moiety, which accommodates the S1 pocket accounted for hydrophobic interactions with Tyr662, Tyr547, Val711 residues (Table 9) (Figure 20).

Furthermore, it is noticed that Glu206 is interacting with N-atom attached to the adamantane ring forming a 2H-bond, whereas Glu205 and Tyr631 are founded to be forming a single H-bond with the respective residue. In Figure 21, The XP visualization of sitagliptin illustrates hydrophobic atoms in a ball and stick form (shown in a thick green hue), and the hydrophobic atoms on the protein that bounds the hydrophobic group of the ligand is shown in a grey CPK representation. The hydrophobic amino acids of DPP-4 are labeled with a brown color. In the given image, the hydrophobic section of sitagliptin is surrounded by amino acids such as Tyr662, Val711, Val656, Tyr666, Phe357, Tyr631, Tyr547, and Trp659. The eight docked compounds revealed that Glu205, Glu206, and Tyr547 are the most frequent amino acids that formed an H-bond against DPP-4 protease.

## 7. Future Perspectives

DPP-IV is an attractive target for the development of compounds to treat T2DM. Numerous DPP-4 inhibitors are in the advanced stages of clinical development. The use of DPP-4 inhibitors (DPP-4i) as a therapeutic strategy for the treatment of T2DM has increased significantly over the past two decades. DPP-4i, also known as dipeptidyl peptidase-4 inhibitors, are a class of drugs that inhibit the enzyme dipeptidyl peptidase-4, which is involved in the degradation of incretin hormones. Incretin hormones are responsible for increasing insulin production in response to food intake, and thus, by inhibiting their degradation, DPP-4i can improve glycemic control [73,74]. The development of novel and highly effective compounds based on scaffolds possessing halogen groups has been a growing area of interest in research related to developing DPP-4 inhibitors. Halogenated DPP-4 inhibitors have the potential to be more effective and safer than existing treatments for diabetes because they can be better targeted to the enzymes involved in the disease. Halogenated scaffolds have been found to possess several advantages, such as improved solubility, enhanced pharmacokinetic properties, and improved metabolic stability. These advantages make halogenated scaffolds a promising platform for developing new DPP-4 inhibitors [67,75]. Halogenated compounds are more hydrophobic, which could enable them to penetrate the cell membrane more quickly and effectively than existing treatments. This could improve the drug’s efficacy and reduce the amount of drug needed for a successful treatment. In addition, halogenated DPP-4 inhibitors could also lead to the development of new synthetic routes that are more cost-effective. The use of halogenated scaffolds for developing DPP-4 inhibitors is likely to continue to be an area of increasing interest in the future [75,76,77]. In particular, halogenated scaffolds with different chemotypes, such as amides, sulfonamides, and thiazoles, may offer new opportunities for developing more potent and selective molecules. In addition, using halogenated scaffolds in combination with other chemotypes, such as macrocycles, may also offer new possibilities for developing more potent and selective DPP-4 inhibitors. In addition, using different chemotypes, such as amides, sulfonamides, and thiazoles, and the combination of halogenated scaffolds with other chemotypes, such as macrocycles, may further expand the possibilities for the development of more potent and selective DPP-4 inhibitors [77,78]. By utilizing novel synthetic pathways, drug manufacturers could produce more affordable medications. This could benefit patients who no longer have to pay expensive out-of-pocket costs for their treatment. Overall, using DPP-4 inhibitors with halogenated scaffolds and efficient schemes could lead to more effective drugs for treating T2DM. Recently, several novel DPP-4 inhibitors incorporating halogenated scaffolds have been developed, and these compounds have demonstrated promising pharmacological activity in vitro and in vivo. For instance, a series of new DPP-4 inhibitors containing halogenated benzene and cyclohexane derivatives demonstrated significant inhibition of DPP-4 in human plasma in a dose-dependent manner. Additionally, these compounds displayed good oral bioavailability and excellent metabolic stability. Recent studies have focused on developing new scaffolds for DPP-4i containing halogen groups. Halogens have been shown to increase the potency of DPP-4i by providing additional interactions with the enzyme and thus to enhance their inhibitory activity [79,80]. This is particularly evident in the case of fluorine-containing DPP-4i, which exhibit up to 10-fold higher inhibitory activity than their non-halogenated analogs. In addition to halogenated DPP-4i, there is also increasing interest in developing DPP-4i that encircle highly efficient schemes. These compounds are designed to interact simultaneously with various binding sites on the enzyme. This type of structure allows for improved binding affinity and selectivity and may also increase the drug’s potency. The future of DPP-4i with halogenated scaffolds and highly efficient schemes looks promising. Further research is needed to optimize these compounds’ synthesis and assess their safety and efficacy in clinical trials. Additionally, the development of new DPP-4i scaffolds and schemes is ongoing, and the use of computational techniques to design and evaluate novel compounds is becoming increasingly important. These compounds exhibited excellent bioavailability and outstanding metabolic stability in vitro and in vivo. The future of halogenated scaffolds for the development of DPP-4 inhibitors is promising. With further exploration and optimization of these scaffolds, more selective, potent, and bioavailable DPP-4 inhibitors can be developed [81,82]. Other studies are needed to evaluate these compounds’ pharmacological and toxicological profiles. Additionally, developing advanced computational techniques, such as molecular modeling and quantitative structure-activity relationship (QSAR) analysis, could facilitate the design and optimization of these scaffolds. In conclusion, developing novel DPP-4 inhibitors based on halogenated scaffolds is a promising area of research. These compounds have shown good pharmacological activity and metabolic stability in vitro and in vivo. With further optimization of these scaffolds, more selective, potent, and bioavailable DPP-4 inhibitors can be developed [78,81]. In the future, the use of halogenated DPP-4 inhibitors could provide a promising new approach to the treatment of T2DM.

## 8. Conclusions

The development of novel DPP-4 inhibitors is of great interest to researchers due to their potential to improve bioavailability, potency, and pharmacokinetic and pharmacodynamic properties. Scaffolds are an essential component of drug design, and various novel DPP-4 inhibitors have been developed using diverse scaffolds, some of which are currently undergoing clinical trials. This review will provide an overview of the multiple scaffolds used for creating DPP-4 inhibitors and the SAR of the compounds developed. The use of halogen atoms in synthesizing DPP-4 inhibitors is more efficient than using methoxy, C-chain, cyano, and other heteroatoms. Halogen atoms are electronegative; thus, they bind more efficiently with DPP-4 while also providing hydrophobic properties to the compound. Furthermore, tri-fluorophenyl and adamantane moiety have been used to design novel DPP-4 inhibitors using several other scaffolds such as tetrahydropyran, pyridine, piperidine, morpholine, 1,2,3-triazole, xanthine, a thiazolidinedione, pyrrolidine, and pyrazole.

SAR defines the role of each substitution of atoms including hydrophobic etc. which also helps us in understanding the biological activity, binding affinity, interaction with residues against DPP-4 and also plays a vital role in drug design, synthesis of moiety based compounds. 3D and 2D interaction images of marketed various marketed DPP-4 inhibitors have been illustrated. Various schemes of gliptins have been discussed encircling high efficient yield. In addition to using various scaffolds, researchers are also exploring usingvarious”synthesis” schemes to develop novel DPP-4 inhibitors. This involves reducing the number of steps and chemicals used in the synthesis, thereby reducing the amount of aqueous waste generated. This technique is also beneficial in increasing the yield of the compounds. Numerous challenges in development of DPP-4 inhibitors have been seen such as % yield of synthesized compounds, gastrointestinal problems, skin reactions and majorly high risk of developing pancreatitis have been observed experiencing severe pain in upper abdomen. To conclude, developing novel DPP-4 inhibitors is an ongoing endeavor for researchers. The various scaffolds and SARs of compounds used in the synthesis of DPP-4 inhibitors will be of great help to the scientific community. Additionally, the use ofvarious” Synthesisschemes” techniques can further help in the development of potent compounds with extreme biological activity.

## Figures and Tables

**Table 1:** Illustrating type of class interacting at which subsite of DPP-4 protease.

S.No	Class of Drug	DPP-4 Inhibitor Drugs	Interaction Subsite of DPP-4	Brief Detail
1.	First	Sitagliptin and teneligliptin	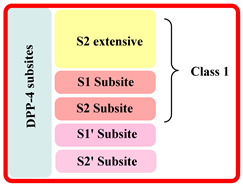	▪ Teneligliptin has 5 times higher activity than sitagliptin because of the presence of a J-shaped anchor-lock domain and stronger covalent bond with DPP-4, also an additional bond with S_2_ extensive subsite.
2.	Second	Vildagliptin and saxagliptin	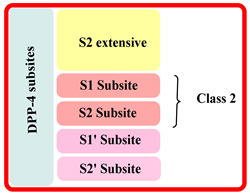	▪ Cyanopyrrolidine scaffold binds to S_1_. ▪ Hydroxyadamantyl group binds to the S_2_ subsite. ▪ Saxagliptin has five-times higher activity than vildagliptin.
3.	Third	Alogliptin and linagliptin	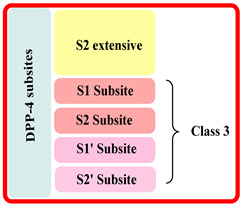	▪ Alogliptin binds to S_1_, S_2_ and S_1_′ subsites. ▪ Linagliptin binds to S_1_, S_2_, S_1_′, S_2_′ subsites. ▪ Compared with alogliptin, Linagliptin has 8-fold higher activity.

**Table 2:** IC_50_ of various derivatives synthesized compound (**10**).

S. N.	Compounds	DPP-4 IC_50_ (nM)	Types of Interaction
1.	**10A**	14.71 ± 0.82	2 H-bond interacting with Arg125 and Tyr547
2.	**10B**	2.19 ± 0.14	3 H-bond interacting with Arg125 1 H-bond interacting with Tyr547 1 π-bond interacting with Tyr547
3.	**10C**	1.42 ± 0.11	3 H-bond interacting with Arg125 1 H-bond interacting with Tyr547
4.	**10D**	0.51 ± 0.03	2 H-bond interacting with Lys554 and Glu206
5.	**10E**	0.66 ± 0.04	2 H-bond interacting with Tyr547 and Glu206 2 H-bond interacting with Arg125

**Table 3:** 4,6-disubstituted tetrahydro-pyrido[4,3-d] pyrimidine derivates DPP-4 inhibitory activities.

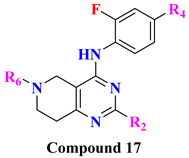					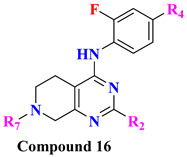				
Comp.	R_2_	R_4_	R_6_	DPP-4 Inhibition (%Inhibition-10 µM)	Comp.	R_2_	R_4_	R_7_	DPP-4 Inhibition (%Inhibition-10 µM)
**17A**	Me	CN	A5	54.3	**18A**	H	Ms	A2	75.8
**17B**	Me	Ms	A2	83.2	**18B**	H	Ms	A7	77.9
**17C**	Me	Ms	A3	52.4	**18C**	Me	CN	A2	74.5
**17D**	Me	Ms	A6	65.1	**18C**	Me	Ms	A2	72.5

**Table 4:** In vitro DPP-4 inhibitory activity of derived derivatives.

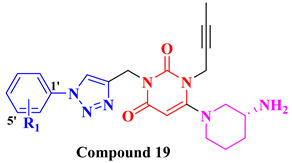			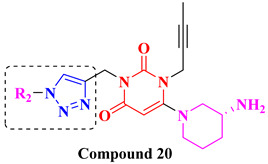		
Compounds	R_1_	DPP-4 Inhibition (nM)	Compounds	R1	DPP-4 Inhibition (nM)
**19A**	H	185.24	**20A**	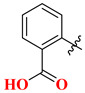	65.63
**19B**	2′-F	64.05	**20B**	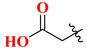	84.72
**19C**	4′-F	135.45	**20C**	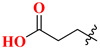	12.45
**19D**	2’,4′-diF	243.67	**20D**	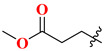	64.31
**19E**	3′-Cl	168.63	**20E**	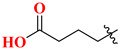	26.81
**19F**	3′-MeO	88.53	**20F**	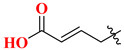	9.56

**Table 5:** Inhibitory activity of target compounds against DPP-4.

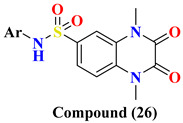		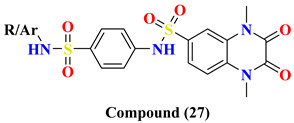	
Compound Number	Ar =		IC_50_ nM
**26A**	-2-Thizolyl		ND
**26B**	-C_6_H_5_-4-COCH_3_		1.28 ± 0.099
**26C**	-C_6_H_5_-4-COOH		0.74 ± 0.103
**26D**	-C_6_H_5_-4-F		0.70 ± 0.112
**26E**	-C_6_H_5_-4-Br		0.71 ± 0.075
**26F**	-C_6_H_5_-2-SH		0.67 ± 0.055
**27A**	H		0.60 ± 0.086
**27B**	-2-Thizolyl		0.93 ± 0.128
**27C**	-2-Pyridinyl		0.48 ± 0.052
**27D**	-2-Pyrimidinyl		0.48 ± 0.050
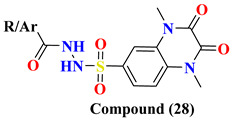		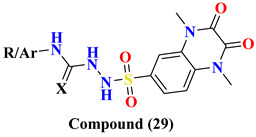	
**28A**	-CH_3_		0.085 ± 0.004
**28B**	-CH_2_Cl		0.095 ± 0.006
**28C**	-C_6_H_5_		0.095 ± 0.012
**29A**	X; O, -CH(CH_3_)_2_		0.039 ± 0.042
**29B**	X; O, -CH_2_-C_6_H_5_		0.048 ± 0.067
**29C**	X; O, -CO-C_6_H_5_		0.144 ± 0.107
**29D**	X; O, -C_6_H_5_-4-Cl		ND
**29E**	X; S, -C_2_H_5_		0.068 ± 0.044
**29F**	X; S, -C_6_H_11_		0.055 ± 0.110
**29G**	X; S, -C_6_H_5_		0.049 ± 0.031
**29H**	X; S, -C_6_H_5_-4-OCH_3_		ND

**Table 6:** DPP-4 inhibitory results of developed analogs of β-amino carbonyl, and 1,2,4-triazoles.

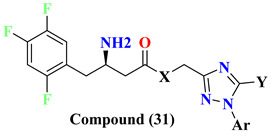				
Compounds	X	Y	Ar	DPP-4 IC_50_ (nM)
**31A**	O	H	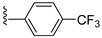	775 nM
**31B**	NH			34.4 nM
**31C**	O	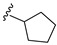	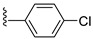	80.3 nM
**31D**	NH			131 nM
**31E**	O	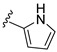	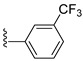	49.9 nM
**31F**	NH			99.8 nM
**31G**	O	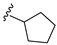	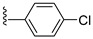	81.6 nM
**31H**	NH			119 nM
**31I**	O	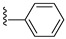	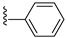	91.3 nM
**31J**	NH			50.4 nM
**31K**	O	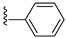	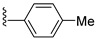	497 nM
**31L**	NH			138 nM

**Table 7:** DPP-4 inhibitory activity of several marketed compounds.

IC_50_ (nM) of Several Marketed Drugs			
Fluorophenyl-based scaffold drugs		Adamantane-based scaffold drugs	
Sitagliptin	Omarigliptin	Vildagliptin	Saxagliptin
18 nM	1.6 nM	2.3 nM	26 nM
Other marketed DPP-4 marketed inhibitors			
Alogliptin	6.5 nM	Teneligliptin	1 nM

**Table 8:** Different scaffold moieties with IC_50_ values.

S.N.	Structure of Compound	Scaffold	IC_50_	Ref.
1	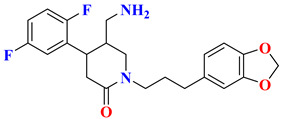	Fluorophenyl-piperidine based	8.5 nM	[29]
2	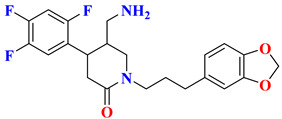	Fluorophenyl-piperidine based	19 nM	[29]
3	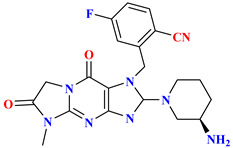	Tricyclic scaffold	1.7 nM	[49]
4	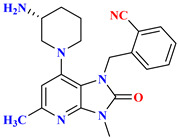	Based on the Alogliptin scaffold (Scaffold hopping)	9 nM	[67]
5	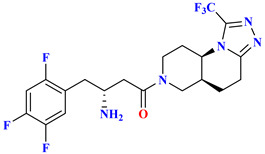	Based on Sitagliptin scaffold (Scaffold hopping)	28 nM	[59]
6	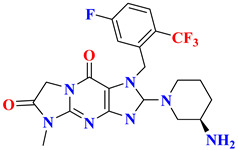	Tricyclic scaffold	11.2 nM	[49]

**Table 9:** Illustrating the type of interaction of Marketed drugs with protein residues.

S.N.	Compounds	Structure	Residue	Interaction
1.	Omarigliptin	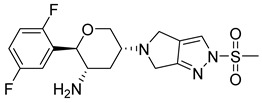	GLU205	H-bond [NH..C=O]
			GLU206	H-bond [C=O..NH and OH..NH]
			TYR666	π-bond [C..C]
2.	Sitagliptin	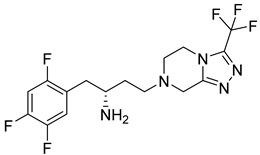	GLU205	H-bond [O..NH]
			GLU206	H-bond [OH..NH and NH..C=O]
			TYR662	H-bond [O..NH]
3.	Teneligliptin	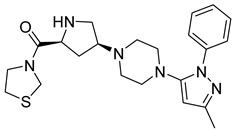	GLU205	H-bond [OH..NH]
			GLU206	H-bond [OH..NH]
			PHE357	π-bond [C..C]
4.	Anagliptin	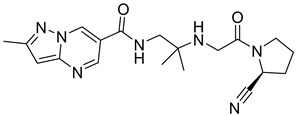	GLU205	H-bond [OH..NH]
			GLU206	H-bond [NH..C=O and NH..OH]
			TYR547	H-bond and π-bond
5.	Linagliptin	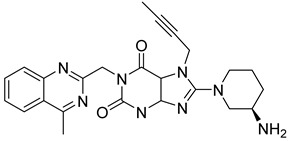	GLU205	H-bond [OH..NH]
			GLU206	H-bond [OH..NH and C=O..NH]
			ARG125	π-cation bond [NH..CH]
			TYR547	π-bond [C..C]
			TYR631	H-bond [C=O..NH]
6.	Saxagliptin	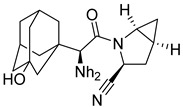	GLU205	H-bond [OH..NH]
			GLU206	H-bond [NH..C=O]
			TYR662	H-bond [NH..C=O]
			TYR547	H-bond [OH..OH]
			ARG669	H-bond [NH..OH]
			ASN710	H-bond [NH..OH]
7.	Alogliptin	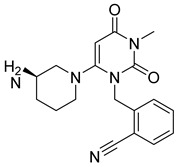	GLU205	H-bond [NH..C=O]
			GLU206	H-bond [NH..OH]
			TYR666	π-bond [C..C]
			TYR547	H-bond [N..OH]
8.	Vildagliptin	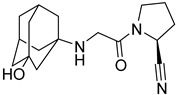	GLU205	H-bond [C=O..OH]
			GLU206	H-bond [C=O..NH] and [OH..NH]
			TYR631	H-bond [N..NH]

## Data Availability

Not applicable.

## Abbreviations

T2DM	Type-2 diabetes mellitus
DPP-4	Dipeptidyl peptidase-4
SAR	Structure activity relationship
GLP-1	glucagon-like peptide-1
GIP	Glucose-dependent insulinotropic polypepide
DM	Diabetes mellitus
IC_50_	inhibitory concentration
cAMP	Cyclic adenosine monophosphate
BP	Blood pressure
GPCR	G-protein coupled receptors
MOE	Molecular operating environment
Nm	Nanomolar
NaH &MsCl	sodium hydride and methanesulfonyl chloride
MeCN	methyl cyanide
TEA	triethyl amine
EDC	1-ethyl-3-(3-dimethylaminopropyl)carbodiimide
PrOH	Propyl alcohol
FDA	Food and drug administration
H-bond & OH	Hydrogen bond and hydroxy
COOH	carboxlic acid
EDG withEWG	Electron donating group with Electron withdrawing groups
µM	Micro molar
HIV	Human immunodeficiency viruses
XP	Extra precision
